# JCAD-Derived from Plasma Exosomes: Promoting Tumor Cell Progression and Predicting Poor Outcomes in Breast Cancer

**DOI:** 10.7150/ijbs.107763

**Published:** 2025-07-25

**Authors:** Douwaner Liu, Min Xiong, Xiaoting Chen, Xuliren Wang, Yuting Sang, Shiyang Liu, Liyi Zhang, Weiru Chi, Hengyu Ren, Bingqiu Xiu, Qi Zhang, Yayun Chi, Jiong Wu, Jingyan Xue

**Affiliations:** 1 Key Laboratory of Breast Cancer in Shanghai, Department of Breast Surgery, Fudan University Shanghai Cancer Center, Shanghai 200032, China.; 2 Department of Radiation Oncology, Huadong Hospital, Fudan University, Shanghai 200040, China.; 3 Department of Oncology, Shanghai Medical College, Fudan University, Shanghai 200032, China.

**Keywords:** Breast cancer, Exosome, JCAD, Wnt/β-Catenin pathway

## Abstract

**Background:** Breast cancer has the highest incidence among all cancers in women, and the prognosis of breast cancer is strongly linked to the stage of the disease. As one of the components found in liquid biopsy samples, exosomes are membranous vesicles that are actively secreted by living cells. Therefore, the key genes in exosomes may serve as biomarkers for predicting the prognosis of breast cancer patients.

**Methods:** In this study, 128 blood samples collected from breast cancer patients at Fudan University Shanghai Cancer Center between June 2018 and March 2019 were subjected to transcriptome sequencing, and the resulting dataset was used as the training dataset. A LASSO regression model was employed for screening prognostic genes. Additionally, 131 patient samples from February 2020 to February 2022 were collected to establish the validation dataset. The corresponding phenotypes and mechanisms of the key genes were confirmed by CCK8 cell proliferation, colony formation, EdU cell proliferation, flow cytometry, transwell cell migration, scratch assay, animal study and RNA-seq assays.

**Results:** Eleven differentially expressed genes tended to increase from the benign stage to the late stage of breast cancer. Five genes were further identified by LASSO regression analysis to establish a prognostic model. The time-dependent receiver operating characteristic (ROC) curves revealed area under the curve (AUC) values of 0.858 for the 1-year follow-up and 0.772 for the 2-year follow-up. The time-dependent ROC curve of the validation dataset indicated an AUC value of 0.840 for the 1-year follow-up. JCAD, a gene closely associated with prognosis, was selected for further investigation. The experimental results demonstrated that JCAD may activate the Wnt/β-catenin pathway by increasing FZD1 expression, thereby promoting the EMT process and breast cancer progression.

**Conclusions:** Exosomal JCAD, as a prognostic marker, plays an important role in the diagnosis and treatment of breast cancer.

## Introduction

Breast cancer accounts for approximately one-third of all cancer cases 1. The prognosis of breast cancer is highly stage dependent, with the 5-year survival rate decreasing from 95% to 30% as the disease progresses from early to advanced stages 2. Thus, identifying prognostic biomarkers for breast cancer can aid in predicting outcomes and guiding treatment strategies. At present, several biochemical biomarkers, including proteins (HER2, ER, and Ki-67), mRNAs (ERα, ERβ, and ERRγ), and enzymes (CEA and TSGF), are currently used in breast cancer diagnosis. Recent studies have also identified multiple microRNAs, such as miR-21^3^, miR-10b 4, miR-155 5, and miR-145-5p 6, as potential prognostic biomarkers.

Liquid biopsy, which is the collection of bodily fluids, such as blood, urine, saliva, feces, and cerebrospinal fluid 7, 8 for further analysis, is a promising method for diagnosing and monitoring tumor development, as well as predicting cancer prognosis and treatment response 9-13. Liquid biopsies are less invasive than traditional biopsies, and they allow for quicker recovery, enable the study of tumor heterogeneity, and permit repeated testing 10. Exosomes, ranging from 30-150 nm 14, are abundant in bodily fluids, especially blood, and they carry specific biomarkers indicative of their cell of origin, including DNA, RNA, lipids, and proteins 15-17. Exosomes play a role in various stages of cancer progression, including proliferation, migration, invasion, angiogenesis, drug resistance, and epithelial‒mesenchymal transition (EMT) 18-22. The dynamic secretion of exosomes reflects the behavior of living cells, making their contents valuable for cancer diagnosis and prediction 23.

Metastasis, which is driven primarily by EMT, is a significant factor in the poor prognosis of breast cancer 24. EMT is a process in which cells lose their epithelial characteristics and acquire mesenchymal properties, promoting cell migration and activating cancer cells 25, 26. This reversible phenomenon allows mesenchymal cells to revert to epithelial cells or other types. By transforming tightly bound epithelial cells into highly mobile mesenchymal cells, EMT facilitates cancer cell invasion 27.

Here, we aimed to develop a prognostic prediction model for breast cancer patients by performing liquid biopsy transcriptome sequencing. The accuracy of the model was rigorously validated in an independent cohort. We also identified Junctional cadherin 5-associated (JCAD) as a key gene that influences validated phenotypes and investigated its underlying mechanisms. Specifically, we focused on how JCAD activates the EMT signaling pathway, thereby facilitating tumor progression.

## Materials and Methods

### Clinical Samples

Whole blood samples were collected from patients visiting the breast surgery department of Shanghai Cancer Center from June 2018 to March 2019 and from February 2020 to February 2022. Pathological biopsy identified the breast tumors as benign or malignant, and the clinical stages of the breast tumors were determined before treatment. The study cohort consisted of 128 patients from the first period, and the validation cohort consisted of 131 patients from the second period. All enrolled patients were female, and the collection of clinical was were approved by the Ethics Committee of Fudan University Shanghai Cancer Center.

### Cell Culture

MDA-MB-231, BT549, LM2, and HEK-293T cells were cultured in high-glucose DMEM supplemented with 10% fetal bovine serum and 1% triple antibiotic (complete culture medium). After reaching confluence, the cells were passaged.

### Bioinformatics

The bioinformatics analysis was conducted using R Studio (R x64 version 4.1.0). The plasma exosomal RNA samples were subjected to high-throughput sequencing by Anshengda (Nanjing) Life Science Technology Co., Ltd., using the Illumina platform. The sequencing results were exported as count values, and batch effects were removed using the sva package in R Studio, excluding genes with over 10 zero counts. Differential expression analysis between Stage I and Stage IV patients was performed by the DESeq2 and tidyverse packages in R. The FDR method was used to correct p values (Adj. P), with differential gene screening criteria set at log2FoldChange > 1 and Adj. *P* < 0.05. Differential gene heatmaps, volcano plots, and principal component analysis (PCA) maps were created using the pheatmap, ggrepel, and ggfortify packages in R. Genes with CountsMean >500 among the upregulated differentially expressed genes were selected for further analysis 28-30. LASSO regression analysis was performed using the glmnet and survival packages in R, setting the random number of seeds to 2 and the number of cycles to 1000. The LASSO regression model successfully identified key prognostic genes and coefficients. The risk score was calculated using the following formula: score = ∑ (coefi × Expri); where Expr is the expression level; and coef is the corresponding coefficient of each prognostic gene.

### Western Blot Analysis

SDS-PAGE gels were prepared according to the required protein sample concentration. The electrophoresis device was assembled, and 1× electrophoresis solution was added to the tank. Protein samples and markers were loaded into the wells, and electrophoresis was run at 70 V, followed by 110 V after band separation. The PVDF membranes were activated with methanol, and the membrane transfer device was assembled. Membrane transfer was conducted at 250 mA, with the time adjusted according to protein size. The PVDF membranes were blocked with 5% skim milk in TBST for 1 hour and then washed with 1× TBST three times for 10 minutes each. The membranes were incubated with primary antibodies overnight at 4 ℃, followed by three 10-minute washes with 1× TBST. The samples were incubated with secondary antibodies for 2 hours, followed by three 15-minute washes with 1× TBST. ECL working solutions A and B were mixed for exposure, and photos were taken. Related antibody information could be found in **Supplementary Table 1**.

### Reverse Transcription of RNA and qRT‒PCR

Reverse transcription of the plasma extracellular RNA was performed using the TaKaRa PrimeScript™ RT Master Mix and the TaKaRa External Standard Kit (λ polyA) for qPCR. Reverse transcription of cellular RNA was completed using the HiScript III 1st Strand cDNA Synthesis Kit (+gDNA wiper). qRT‒PCR was performed using the ChamQ SYBR qPCR Master Mix Kit, and the relevant qRT-PCR primers are shown in **Supplementary Table 2**. Besides, PCR primers could be found in **Supplementary Table 3**.

### Extraction of Exosomes

Plasma exosomes were extracted using the exoRNeasy Serum/Plasma Midi Kit. For extracellular vesicles, when the cell density reached 70-80%, the culture medium was replaced with serum-free medium. After 48 hours, the supernatants were collected and centrifuged at 2,000 × g for 10 minutes at 4 ℃. The supernatant was then centrifuged at 10,000 × g for 30 minutes at 4 ℃, followed by ultracentrifugation at 110,000 × g for 70 minutes at 4 ℃. The pellet was resuspended in PBS, and ultracentrifugation was repeated. The final pellet was resuspended in 100 μl of PBS, transferred to a 1.5 ml EP tube, and stored at -80 ℃.

### CCK8 Cell Proliferation Assay

The cells were counted and adjusted to a concentration ranging from 8,000-10,000 cells/ml. The outermost wells of a 96-well plate were filled with 100 μl of PBS to reduce evaporation. The cells were evenly distributed into a 96-well plate, with each well containing 200 μl of cell suspension. Each cell type was tested in triplicate. The CCK8 reagent was diluted 1:9, protected from light, and added to each well at a fixed time daily. After 2 hours of incubation, the absorbance at 450 nm was measured using an enzyme-linked immunosorbent assay. Measurements were taken daily for 7 days, and a growth curve was plotted.

### Clone Formation Assay

The cells were counted and adjusted to a concentration of 1,000-2,000 cells/ml. The cells were added to each well of a 6-well plate and incubated undisturbed. After 72 hours, the medium was changed, and cell adhesion was observed under a microscope. The cells were cultured for an additional 2‒3 weeks, with the medium changed every 3‒5 days until clonal patches were visible. The cells were fixed with 2 ml of methanol for 1 hour, stained with 2 ml of 1× crystal violet dye for 30 minutes, washed with PBS, dried, and photographed to count the clones.

### EdU Cell Proliferation 31 Assay

The cell proliferation ability was assessed using the Cell Light EdU Apollo 567 *In vitro* Kit (100T) (RiboBio, C10310-1, Guangzhou, China). Experiments were conducted in a 96-well plate with 1×10^4^-2×10^4^ cells per well. The cell proliferation rate was calculated as the ratio of the number of EdU-incorporated cells to the total number of cells calculated on the basis of DAPI staining of the cell nuclei.

### Cell Apoptosis Flow Cytometry 32 Assay

When the cell density reached 70-80%, apoptosis was induced by replacing the complete culture medium with medium containing paclitaxel at a concentration of 80 nM or docetaxel at a concentration of 40 nM and incubating the cells for 72 hours. The cells were stained with the reagents in the PE Annexin V Apoptosis Detection Kit I (BD, 559763, USA), and flow cytometry was performed to detect cell apoptosis. Unified gating conditions are required within each control group.

### Transwell Cell Migration Assay

The cells were counted and adjusted to a concentration of 5×10^5^-6×10^5^ cells/ml. A 24-well plate was filled with 700 μl of culture medium containing 20% serum. The Transwell chamber was placed in the center of each well, and 200 μl of cell suspension was added to the upper chamber. After appropriate incubation (e.g., 16 hours for MDA-MB-231), the chambers were washed with PBS, fixed in methanol for 30 minutes, stained with 1× crystal violet for 30 minutes, and washed, and the cells from the upper chamber were removed. The chambers were air-dried, and the migrated cells were photographed and counted under a microscope.

### Scratch Assay

LM2 cells were seeded into 6-well plates. Once the cells reached 95-100% confluence, a sterile 200 µl pipette tip was used to make a straight scratch in the cell monolayer. The wound area was recorded at 0, 8, 12 and 16 hours to assess the degree of closure by using a microscope.

### BALB/c Mouse Study

All female BALB/c nude mice (6 weeks of age) were purchased from Shanghai SLAC Laboratory Animal Co., Ltd., and raised in a standard pathogen-free (SPF) environment, each weighing 20 ± 2 g, in the Experimental Animal Center of Fudan University Shanghai Cancer Center. The animal study was conducted under guidelines approved by the Institutional Animal Care and Ethics Committee of Fudan University Shanghai Cancer Center.

### Subcutaneous Orthotopic Implantation

A subcutaneous injection of 1×10^6^ LM2 cells near the nipples of the nude mice was performed, and the tumor size was recorded every 3 days.

### Tail Vein Injection

LM2 cells (3×10^5^) were mixed with 200 µl of PBS and slowly injected into the mice through the tail vein to avoid vein blockage. Starting one month after injection, fluorescence imaging was performed weekly, as described in the subsequent section, to observe lung metastasis in the mice.

### Fluorescence imaging of Lung Metastasis

For the *in vitro* lung fluorescence imaging, the mice were sacrificed, and their lungs were removed, placed on a black background, and imaged using an *In vivo* Optical 3D Imaging System for Animals (Bruker, USA). *In vivo* lung fluorescence imaging was performed by anesthetizing the mice with isoflurane and taking images while they were lying on a black background.

### Immunohistochemistry and Hematoxylin and Eosin (HE) Staining

The tumor tissues from the nude mice were fixed with formaldehyde and sectioned into slices with thicknesses of approximately 4-5 micrometers using a microtome. The sections were stained with the corresponding antibody and observed under a microscope.

The mouse lung sections were placed in a diluted hematoxylin solution for several minutes, followed by immersion in acidic and ammonia solutions for a few seconds each. The sections were then rinsed under running water for 1 hour and then placed in ddH_2_O. The sections were dehydrated by placing them in 70% and 90% ethanol for 10 minutes each. The sections were stained with alcoholic eosin solution for 3 minutes, followed by dehydration with pure alcohol. The sections were made transparent with xylene. After applying mounting medium, and the sections were sealed with a cover slip. The sections were observed and photographed under a microscope.

### RNA-Seq

RNA-seq was performed by Suzhou Genewiz Co., Ltd.

### XAV939 Treatment

XAV939 dry powder (Selleck, USA) was diluted with DMSO to prepare a 20 mM XAV939 stock solution. XAV939 stock solution was then diluted with complete culture medium, and XAV939 working solution was prepared at a concentration of 10 µM. The cells were subsequently cultured in working medium, while a DMSO solution diluted 1:2000 in complete culture medium was used as a control.

### Statistical Methods

The experimental data are expressed as the means ± standard deviations (SDs) and were analyzed using IBM SPSS Statistics 20 (BM Corp, Armonk, NY) or GraphPad Prism 9.0 software (GraphPad Software, San Diego, CA, USA). The experiments were repeated at least 3 times. P values were calculated using theStudent's or Welch's t test for continuous variables and the Pearson chi-square test for categorical variables. Survival analysis was performed by the Kaplan‒Meier method and log-rank tests. Two-tailed P values < 0.05 were regarded as statistically significant. In the figures, the level of statistical significance is indicated as ns (P > 0.05), * (P < 0.05), ** (P < 0.01), or *** (P < 0.001).

## Results

### JCAD Expression in Plasma Exosomes and Breast Cancer Prognosis

Plasma samples were collected from 128 breast cancer patients in Fudan University Shanghai Cancer Center from June 2018 to March 2019. The clinicopathological characteristics of these patients are summarized in Table 1, and there were 26 Stage I, 36 Stage II, 15 Stage III, and 34 Stage IV patients** (Supplementary Fig. 1A-C, Table 1)**. RNA was extracted from plasma exosomes of all patients and subjected to high-throughput sequencing. Among the sequencing results, protein-coding RNA constituted the highest proportion (80.1%), followed by lncRNA, pseudogenes, and small non-coding RNA **(Supplementary Fig. 1D)**. We identified the extracted plasma exosomes. Spherical vesicles can be seen under transmission electron microscopy and scanning electron microscopy. Nanoparticle tracking analysis (NTA) shows that the majority of plasma exosomes have a particle size ranging from 50-100nm, which is consistent with the normal particle size range of exosomes **(Supplementary Fig. 1E-G)**. Through Western Blot, it was found that the protein expression of surface markers (CD63 and CD81) on plasma exosomes could be detected, but the protein expression of GM130, β-Actin, and GAPDH that widely present in cells could not be detected **(Supplementary Fig. 1H)**.

**Figure 1:**
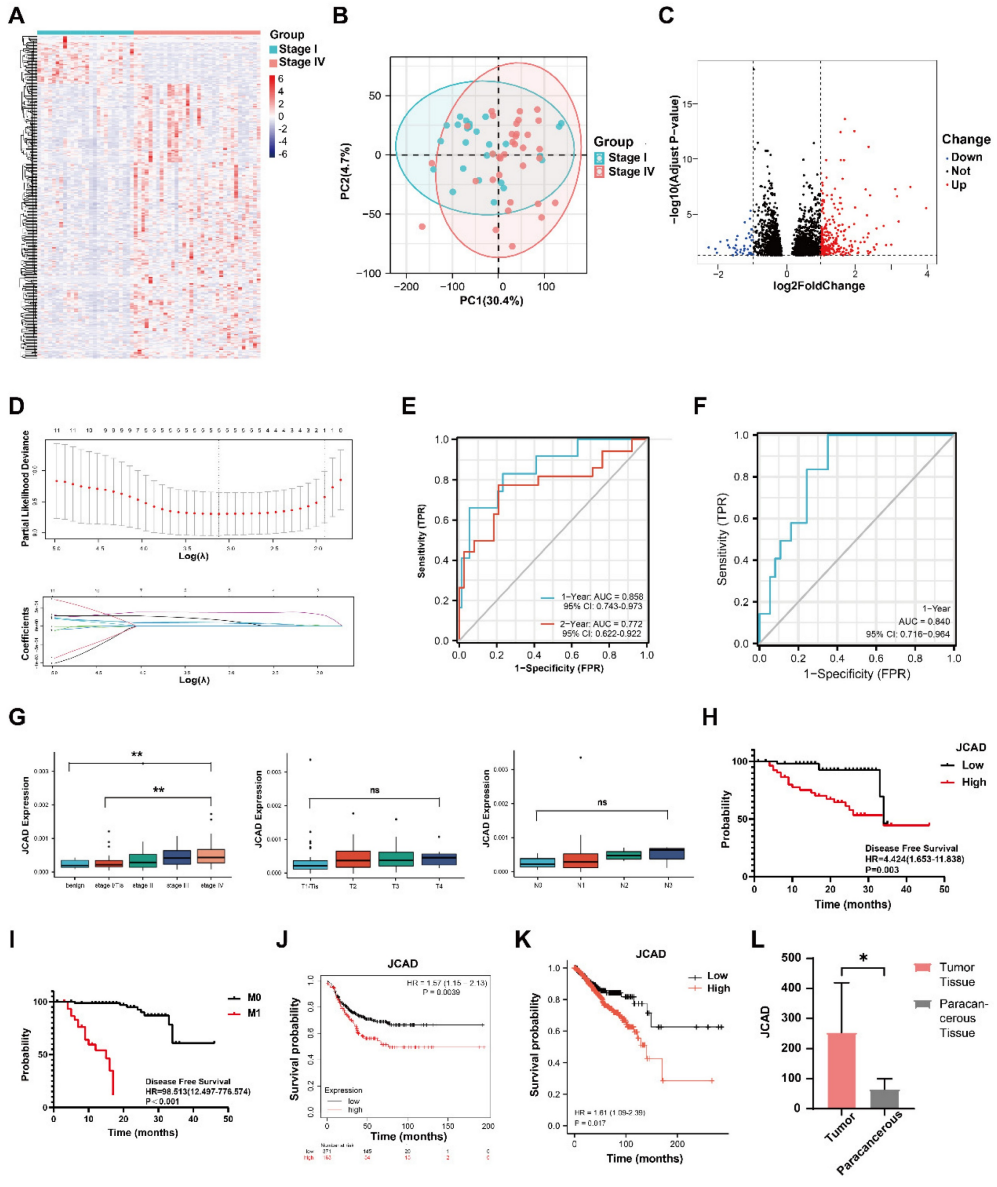
** JCAD is highly expressed in breast cancer patients with poor prognosis. A:** Heatmap of differentially expressed genes in patients with stage I and IV breast cancer. **B:** Principal component analysis (PCA) diagram showing the differences in gene expression between stage I and IV breast cancer patients. **C:** Volcano map of differentially expressed genes in patients with stage I and IV breast cancer. **D:** Change in partial likelihood bias in LASSO regression with log(λ) and the coefficient of the independent variable in LASSO regression with log(λ). **E:** Time-dependent ROC curve of the scores of the five genes related to prognosis in the study cohort. **F:** Time-dependent ROC curve of the scores of five genes related to prognosis in the validation cohort. **G:** Box chart of the expression levels of JCAD in the plasma-derived exosomes of patients with benign breast tumors and breast cancer of different stages (T and N stages) in the cohort. **H:** Kaplan‒Meier PFS analysis curves for the JCAD low-expression and high-expression groups in the study cohort; **I:** Kaplan‒Meier PFS analysis curves for the M0 and M1 stages in the study cohort. **J:** Kaplan-Meier survival curves for the JCAD low-expression and high-expression groups in the Kaplan-Meier Plotter public database. **K:** Kaplan‒Meier survival curves of the JCAD low-expression and high-expression groups in TCGA public database. **L:** The expression level of JCAD in tumor tissues and paracancerous tissues of five patients from Fudan University Shanghai Cancer Center.

To identify genes associated with breast cancer prognosis, differential analysis of the high-throughput sequencing results was conducted between Stage I and Stage IV breast cancer patients. A total of 265 upregulated genes were identified **(Fig. 1A-C)**. After excluding genes with low expression levels, 11 genes whose expression levels increased with cancer stage were identified **(Supplementary Fig. 1I-S)**. LASSO regression analysis of these 11 genes revealed 5 prognosis-related differentially expressed genes and their coefficients **(Fig. 1D, Table 2)** as follows:

Score=7.69x10^-5^×Exp*IGFBP5* + 6.75x10^-6^×Exp*JCAD* + 3.71x10^-4^×Exp*MAP1B* + 1.67x10^-4^×Exp*MGP* + 1.01x10^-4^×Exp*VASH1.*

On the basis of the LASSO regression results, the prognostic scores were calculated, and the time-dependent ROC curves were plotted. The area under the curve (AUC) value for the 1-year follow-up was 0.858 (95% CI: 0.743-0.973), and the AUC value for the 2-year follow-up was 0.772 (95% CI: 0.622-0.922)** (Fig. 1E)**.

To further validate the prognostic potential of these five genes, plasma was collected from 131 patients in Fudan University Shanghai Cancer Center from February 2020 to February 2022, and the exosomes were extracted from the plasma samples of these patients and were considered the validation group **(Table 3, Supplementary Fig. 1T-V)**. qRT‒PCR was utilized to measure and validate the expression of the 5 genes in the extracellular vesicles of this group, and the scores were calculated. Combined with the prognostic follow-up data, the ROC curve for the 1-year follow-up had an AUC value of 0.840 (95% CI: 0.716-0.964)** (Fig. 1F)**. These results indicated that the five-gene model was precise and reliable in both the test dataset and the validation dataset.

The gene expression levels were plotted across different stages, and significant differences were detected in the expression of the five genes between the Stage I and Stage IV groups in the validation cohort** (Supplementary Fig. 2A-D)**. The expression level of JCAD clearly increased with increasing stage. A box plot of the expression levels of the JCAD gene at different T and N stages was constructed. Although the differences in the JCAD expression levels were not statistically significant, there was an increasing trend **(Fig. 1G)**. According to the high-throughput sequencing results, patients were divided into low- and high-expression groups on the basis of the median expression of the five genes. The baseline clinical and pathological information of the JCAD high- and low-expression groups in the study cohort is shown in **Table 4**. Univariate and multivariate Cox regression analyses revealed that JCAD expression and M stage were independent prognostic factors **(Fig. 1H-I)**. Detailed univariate and multivariate Cox regression analyses of clinicopathological factors and DFS in breast cancer patients are provided in **Table 5** and** Table 6.** Kaplan-Meier analysis of TCGA databases revealed that high JCAD expression was associated with poor prognosis in patients with breast cancer** (Fig. 1J-K, Supplementary Fig. 2E-Q)**. To further confirm that JCAD exosomes are secreted by tumor cells, tumor tissues and paracancerous tissues were collected from 5 patients with breast cancer at our center, and the expression of JCAD was detected by high-throughput sequencing. The mRNA expression of JCAD in tumor tissues was greater than that in paracancerous tissues **(Fig. 1L)**. These results indicated that JCAD can be used as a potential prognostic biomarker for breast cancer.

### JCAD Transmission Between Breast Cancer Cells Using Exosomes

**Figure 2:**
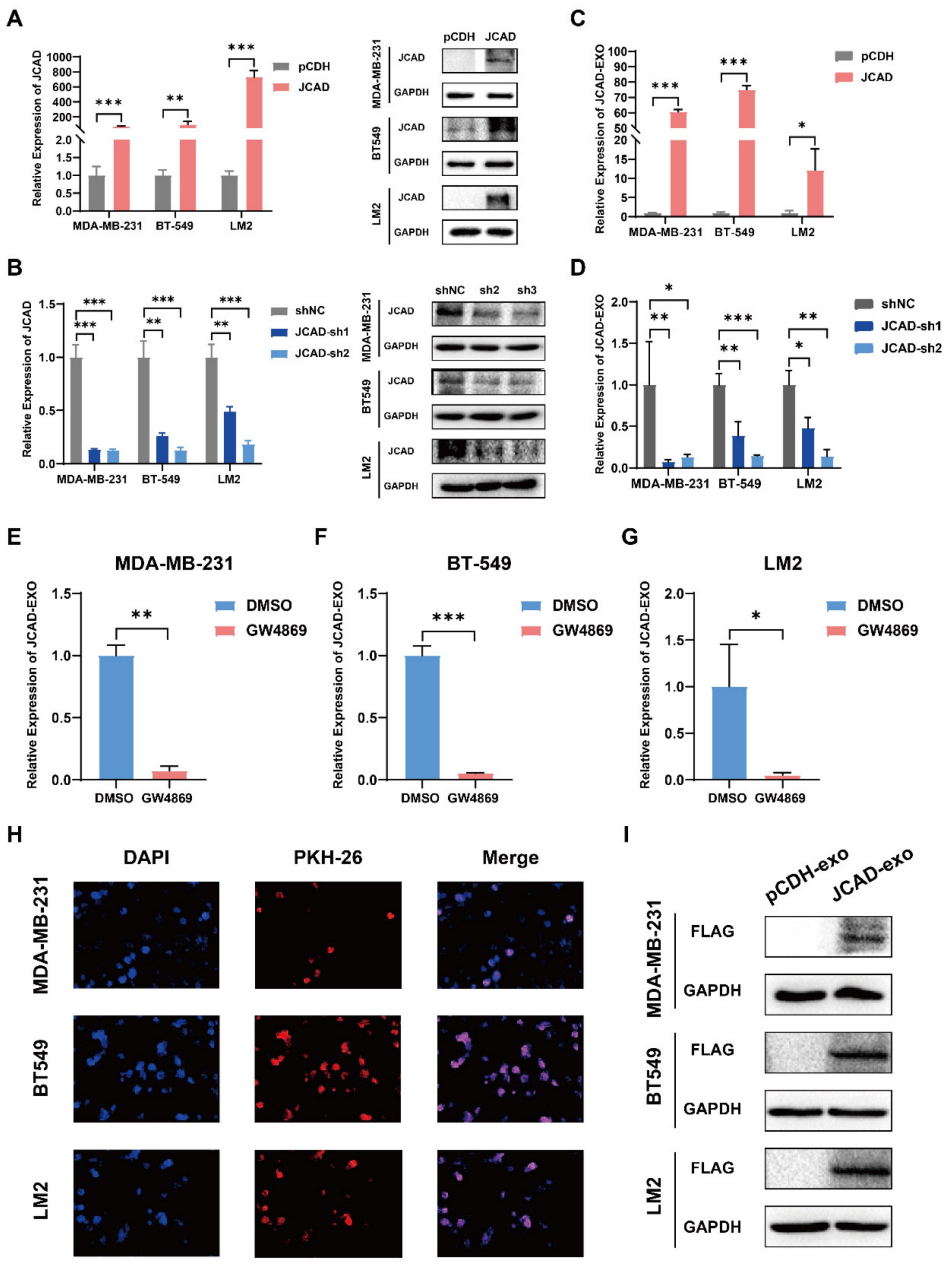
** JCAD is transferred between breast cancer cells through exosomes. A-B:** qRT‒PCR and Western blot analysis of JCAD mRNA and protein levels in stable JCAD-OE and stable JCAD-KD MDA-MB-231, BT549 and LM2 cells and control cells. The y-axis represents the ratio of JCAD to GAPDH expression, with the control set to 1.0. **C-D:** qRT‒PCR analysis of JCAD mRNA levels in exosomes secreted by stable JCAD-OE and stable JCAD-KD MDA-MB-231, BT549 and LM2 cells and control cells. The y-axis represents the ratio of JCAD to proportional λpolyA^+^ RNA-A expression, with the control set to 1.0. **E-G:** The expression level of JCAD in exosomes derived from JCAD-OE MDA-MB-231, BT549, and LM2 cells was inhibited by GW4869. **H:** The exosomes derived from JCAD-OE MDA-MB-231, BT549 and LM2 cells labeled with PKH26 were transferred into the corresponding wild-type cell lines (10×). **I:** After the exosomes derived from JCAD-OE MDA-MB-231, BT549, and LM2 cells were cultured with the corresponding wild-type cell lines, Western blot analysis was used to verify that the Flag protein was detected in the wild-type cell lines.

Compared with luminal breast cancer and Her2-positive breast cancer, triple-negative breast cancer has a poor prognosis and no effective treatment. In this study, three negative breast cancer cell lines with moderate JCAD expression were selected for follow-up research **(Supplementary Fig. 3A-C)**. To further investigate the biological function of JCAD in breast cancer cells, the basal expression of JCAD was overexpressed or inhibited in MDA-MB-231, BT549, and LM2 cells **(Fig. 2A-B)**. To study JCAD at the extracellular vesicle level, extracellular vesicles were extracted from JCAD-overexpressing and JCAD-knockdown cell lines using ultracentrifugation** (Supplementary Fig. 3D)**. The JCAD expression levels in the extracellular vesicles mirrored those in the cells** (Fig. 2C-D)**. When JCAD-overexpressing cells were treated with the GW4869 exosome inhibitor, JCAD expression in the exosomes significantly decreased **(Fig. 2E-G)**. Moreover, RNase A treatment did not significantly alter JCAD expression levels in exosomes **(Supplementary Fig. 3E-G)**, which indicates that JCAD is excreted from tumor cells in the form of extracellular vesicles. In addition, we detected the protein expression of JCAD in exosomes and found that there was no protein form of JCAD in exosomes **(Supplementary Fig. 3H)**. Exosomes derived from JCAD-overexpressing cells were stained with PKH26 and incubated with the corresponding wild-type cells, which resulted in red fluorescence in the wild-type cells **(Fig. 2H)**. Due to the fact that the JCAD overexpression plasmid we constructed is tagged with Flag, the overexpression of JCAD can be reflected by detecting the protein expression of Flag. Western blot validation also showed the presence of Flag protein in wild-type cells **(Fig.2I)**. These findings confirm that JCAD is transmitted between breast cancer cells via exosomes and can be translated into proteins to exert biological functions.

### Exosomal JCAD Promotes the Proliferation and Metastasis of Breast Cancer Cells

**Figure 3:**
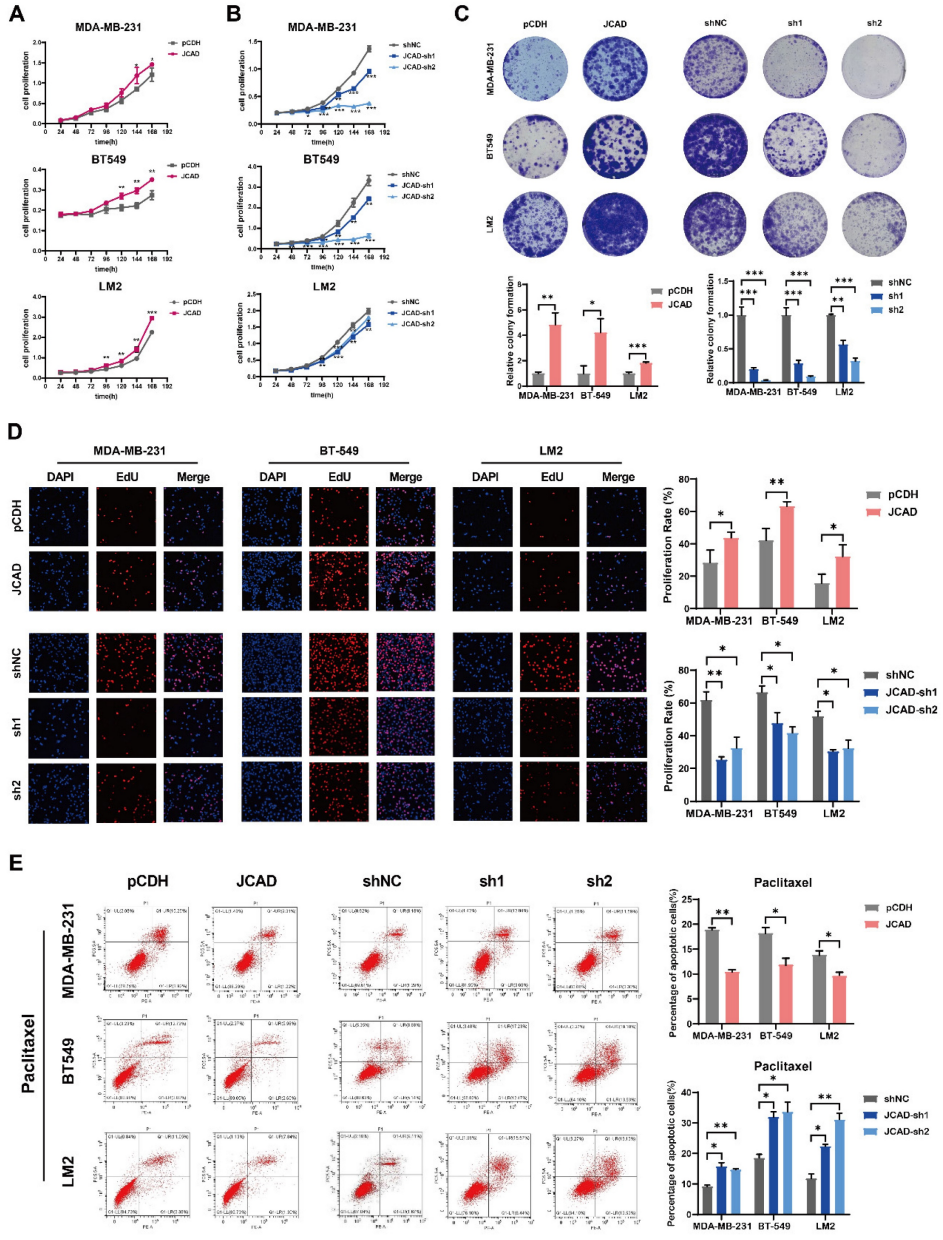
** JCAD promotes the proliferation of breast cancer cells. A-B:** CCK8 cell proliferation experiments were used to verify the proliferation of JCAD-OE and JCAD-KD MDA-MB-231, BT549, and LM2 cells compared with that of control cells. **C:** Clone formation experiments verified the proliferation of JCAD-OE and JCAD-KD MDA-MB-231, BT549, and LM2 cells compared with that of control cells. **D:** EdU cell proliferation experiment verified the proliferation of JCAD-OE and JCAD-KD MDA-MB-231, BT549, and LM2 cells compared with that of control cells (10×).** E:** Induction of apoptosis by adding paclitaxel verified the apoptosis of JCAD-OE and JCAD-KD MDA-MB-231, BT549, and LM2 cells compared with control cells.

**Figure 4:**
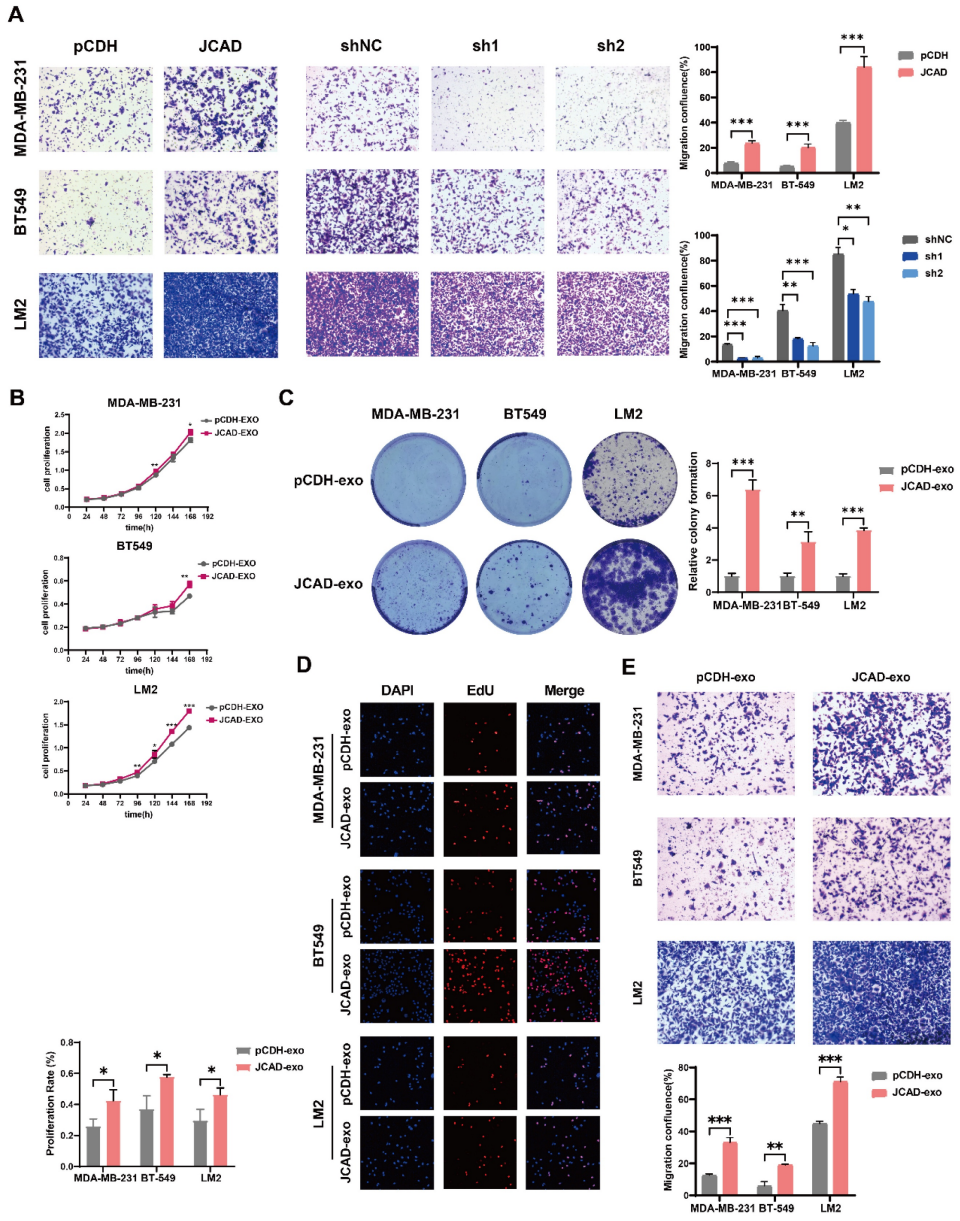
** JCAD promotes the metastasis of breast cancer cells and exosomal JCAD promotes the proliferation and metastasis of breast cancer cells. A:** Transwell cell migration experiments verified the migration of JCAD-OE and JCAD-KD MDA-MB-231, BT549, and LM2 cells compared with that of control cells (10×). **B-D:** CCK8 cell proliferation experiments, colony formation experiments and EdU cell proliferation experiments (10×) verified the proliferation induced by exosomes derived from JCAD-OE and JCAD-KD MDA-MB-231, BT549, and LM2 cells compared with that of control cells. **E:** Transwell migration experiments verified the migration induced by exosomes derived from JCAD-OE and JCAD-KD MDA-MB-231, BT549, and LM2 cells compared with that of control cells (10×).

Compared with control cells, JCAD-overexpressing (JCAD-OE) cells presented greater proliferation, colony formation, and EdU-positive cell ratios whereas JCAD-knockdown (JCAD-KD) cells presented the opposite trend** (Fig. 3A-D)**, confirming the role of JCAD in promoting breast cancer cell proliferation. Flow cytometry analysis revealed that JCAD-OE inhibited the apoptosis induced by paclitaxel and docetaxel and JCAD-KD promoted the apoptosis of breast cancer cells **(Fig. 3E, Supplementary Fig. 4A)**. Transwell cell migration experiment and scratch assay demonstrated that JCAD-OE enhanced breast cancer cell migration, while JCAD-KD inhibits the migration of breast cancer cells. **(Fig. 4A, Supplementary Fig. 4B)**.

Exosomes derived from JCAD-overexpressing cells were added to corresponding wild-type cells, which resulted in enhanced proliferation **(Fig. 4B-D)** and migration** (Fig. 4E)** of breast cancer cells. These findings suggested that exosomal JCAD promotes these processes.

### JCAD Promotes Subcutaneous Tumorigenesis and Lung Metastasis *In Vivo*

**Figure 5:**
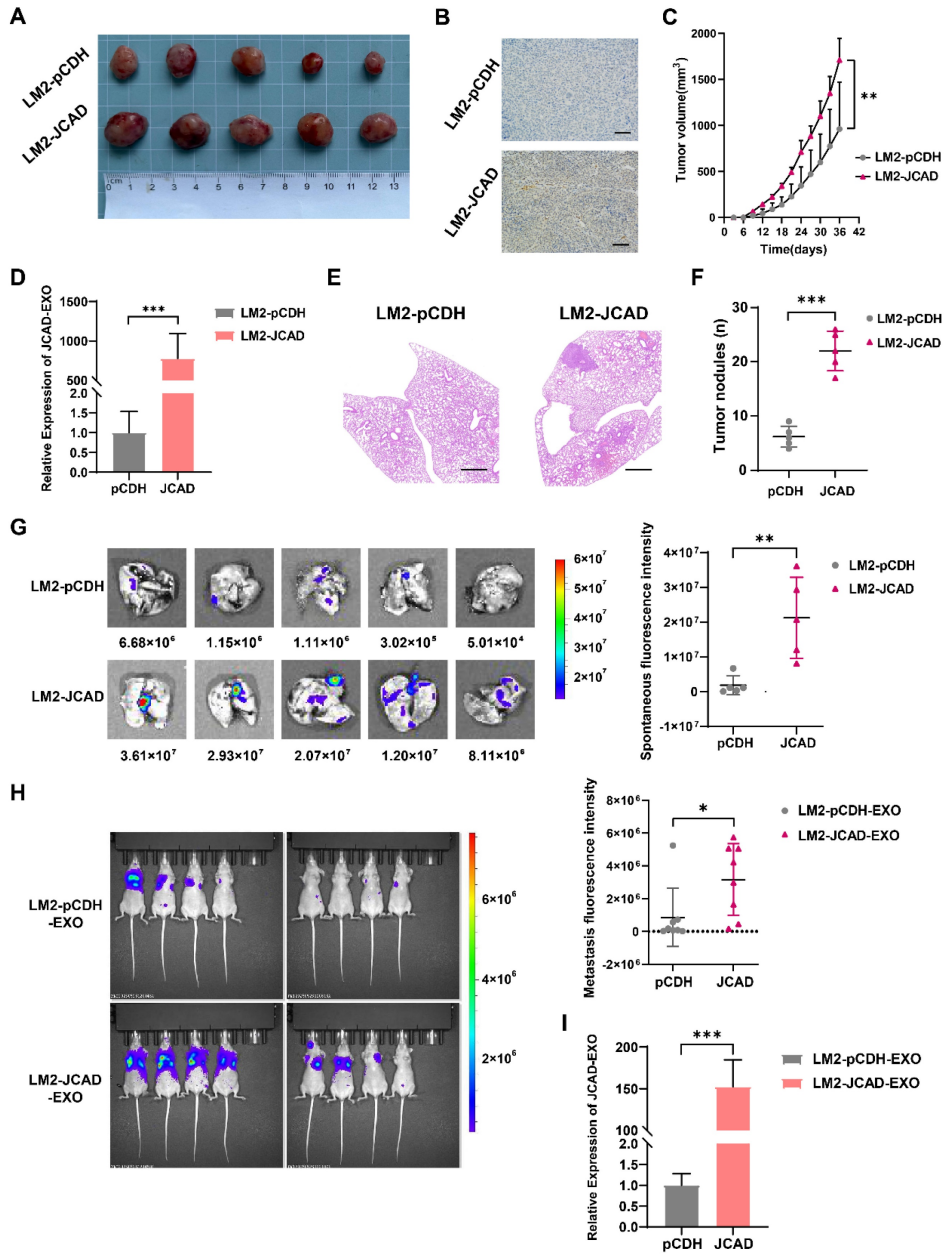
** JCAD promotes subcutaneous tumorigenesis and lung metastasis and exosomal JCAD promotes lung metastasis *in vivo*. A:** Subcutaneous tumor size in the pCDH group and JCAD-OE group.** B:** Subcutaneous tumors from mice in the pCDH and JCAD-OE groups were subjected to JCAD immunohistochemistry (scale bar=100 μm). **C:** Growth curve of tumor volume *in situ* in mice. **D:** RNA expression levels of JCAD in plasma exosomes of the pCDH group and JCAD-OE group. **E:** HE staining of lung metastatic nodules in the two groups of mice (HE: hematoxylin and eosin; scale bar=1000 μm). **F:** Statistics of lung metastatic nodule counts in the two groups of mice. **G:** Spontaneous fluorescence imaging of GFP in lung tissues of the two groups of mice. **H:** Spontaneous fluorescence imaging of GFP in lung tissues of the pCDH-EXO group and JCAD-EXO group with tail vein injection. **I:** RNA expression levels of JCAD in plasma exosomes of the pCDH-EXO group and JCAD-EXO group.

To explore the effects of JCAD on the proliferation and metastasis of breast cancer cells *in vivo*, LM2 cells overexpressing JCAD, which exhibit luciferase/GFP fluorescence, were subcutaneously injected into five pairs of nude mice to establish an *in situ* tumor formation model. Compared with the control, JCAD overexpression significantly promoted *in situ* tumor growth **(Fig. 5A-C)**. At the same time, it was found that the expression of JCAD was higher in the plasma exosomes extracted from mouse eyeballs in the JCAD-OE group **(Fig. 5D)**.

After the mouse lung tissues were excised, nodule counting and fluorescence imaging indicated that both groups experienced lung metastasis. However, compared with the control group, the JCAD-OE group presented significantly greater lung metastasis and an increased number of metastatic nodules **(Fig. 5E-G)**. Thus, these finding suggested that JCAD overexpression in breast cancer cell lines promotes both the proliferation and migration of breast cancer cells.

Next, we also observed the lung metastasis of mice by injecting LM2 cells cultured with JCAD-OE and pCDH exosomes through into the tail vein of eight pairs of nude mice. It was found that mice injected with JCAD-OE exosomes via the tail vein had more and wider lung metastases **(Fig. 5H)**. We also conducted pulmonary nodule counting and confirmed that the JCAD-OE group had more metastatic lung nodules **(Supplementary Fig. 4C-D)**. We tested the plasma exosomes of this batch of nude mice and, similar to *in situ* tumor formation model, also confirmed that JCAD expression was higher in the plasma exosomes of the JCAD-OE group **(Fig. 5I)**.

### JCAD Activates EMT and the Wnt/β-Catenin Pathway and Induces High FZD1 Expression

**Figure 6:**
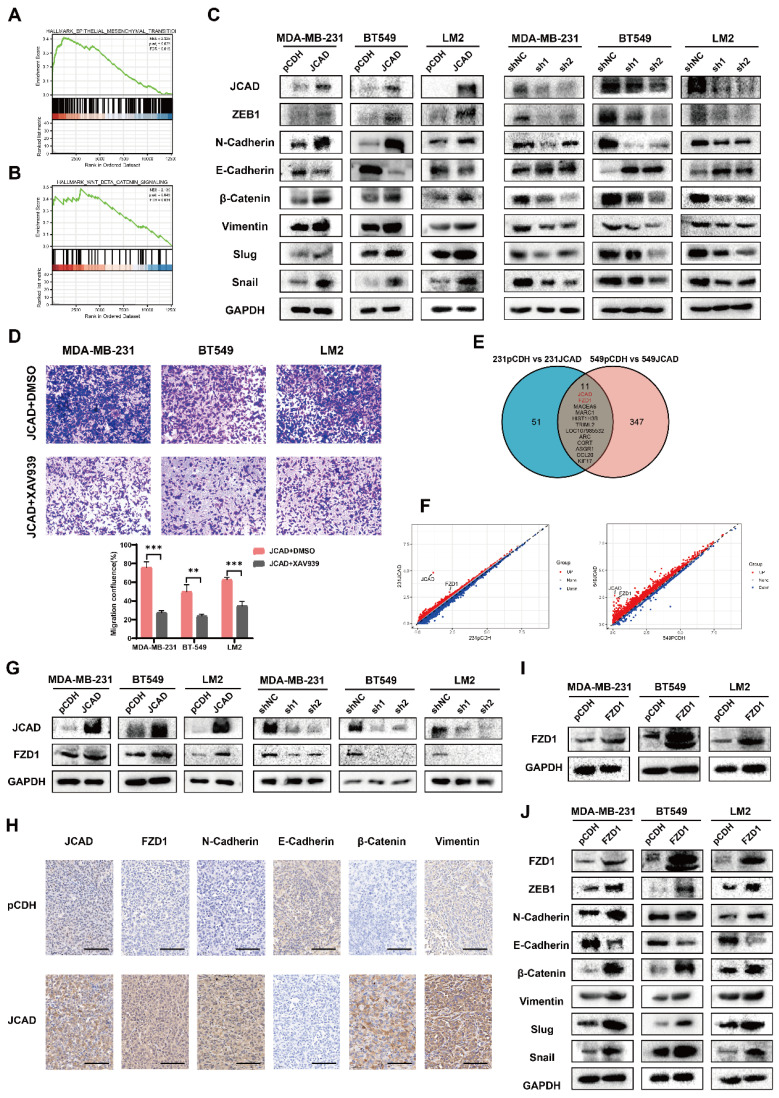
** JCAD activates EMT, activates the Wnt/β-catenin pathway and induces high expression of FZD1. A-B:** Analysis of the RNA-seq results revealed a positive correlation between JCAD and both EMT and the Wnt/β-catenin pathway.** C:** Western blot analysis of the levels of key proteins involved in EMT and the Wnt/β-catenin pathway in JCAD-OE and JCAD-KD cells. **D:** Transwell migration experiments verified that the XAV939 β-catenin inhibitor reduced the migration ability of JCAD-OE MDA-MB-231, BT549, and LM2 cells (10×). **E:** Wayne plot of genes with FC values greater than 2 in the sequencing results of the MDA-MB-231 and BT549 cell lines. **F:** Scatter plot of the sequencing results for the MDA-MB-231 and BT549 cell lines. **G:** Western blot analysis of FZD1 protein levels in JCAD-OE and JCAD-KD cells.** H:** Immunohistochemical staining of key molecules levels of EMT and Wnt/β-Catenin pathway of subcutaneous tumors in pCDH and JCAD-OE groups (LM2) mice (scale=100μm in the figure). **I:** Western blot analysis of FZD1 protein levels in stable FZD1-OE MDA-MB-231, BT549 and LM2 cells and control cells.** J:** Western blot analysis of the protein levels of key molecules involved in EMT and the Wnt/β-catenin pathway in FZD1-OE cells and control cells.

To investigate the downstream mechanisms of JCAD, RNA-seq was performed on JCAD-overexpressing MDA-MB-231 and BT549 cell lines. Joint gene set enrichment analysis (GSEA) analysis revealed a positive correlation with the EMT and Wnt/β-catenin pathways **(Fig. 6A-B)**.

The mRNA and protein expression of EMT-related and Wnt/β-catenin signaling pathway-related molecules were further examined using qRT‒PCR and Western blot analyses. JCAD overexpression increased the mRNA expression levels of N-cadherin, Snail, ZEB1, and β-catenin but decreased the RNA expression levels of E-cadherin. Conversely, knocking down JCAD decreased the mRNA expression levels of N-cadherin, Snail, ZEB1, and β-catenin but increased the RNA expression levels of E-cadherin **(Supplementary Fig. 5A)**. At the protein level, JCAD overexpression activated the EMT and Wnt/β-catenin pathways, whereas JCAD knockdown inhibited the activation of these pathways** (Fig. 6C)**.

JCAD-overexpressing stably transfected cells were treated with the XAV939 β-catenin inhibitor, and tumor cell migration was assessed via a Transwell cell migration assay. Compared with that of the DMSO-treated control group, the migration ability of the XAV939-treated cells was reduced** (Fig. 6D)**. Similar results were obtained in wild-type cell lines cocultured with JCAD-OE exosomes treated with XAV939** (Supplementary Fig. 5B)**. These findings indicated that the XAV939 β-catenin inhibitor counteracts the metastasis phenotype promoted by JCAD and exosomal JCAD.

On the basis of the RNA-seq results, genes with Log2FC values greater than 2 were used to create a Venn diagram. Apart from JCAD, FZD1 ranked highest **(Fig. 6E)**. Scatter plots of the sequencing results for both cell lines revealed significant increases in JCAD and FZD1 expression** (Fig. 6F)**. qRT‒PCR and Western blot analysis confirmed that the overexpression of JCAD increased the RNA and protein expression of FZD1, whereas the knockdown of JCAD decreased FZD1 expression **(Fig. 6G, Supplementary Fig. 6A)**. To further validate, we performed immunohistochemistry on the tumor tissue of mice with subcutaneous *in situ* tumorigenesis shown in Figure 5. The results once again confirmed that the overexpression of JCAD increased the expression of FZD1 and activated the EMT and Wnt/β-catenin pathways **(Fig. 6H)**.

Further detection of mRNA and protein expression levels of EMT-related genes and Wnt/β-catenin pathway members using qRT‒PCR and Western blot analysis revealed that FZD1 overexpression increased the RNA and protein expression levels of N-cadherin, Snail, ZEB1, and β-catenin but decreased the RNA and protein expression levels of E-cadherin, thus activating the EMT and Wnt/β-catenin pathways **(Fig. 6I-J, Supplementary Fig. 6B-C)**. Transwell cell migration assays demonstrated that FZD1 overexpression promoted tumor cell migration, while the XAV939 β-catenin inhibitor counteracted this metastasis-promoting phenotype **(Supplementary Fig. 6D)**.

**Figure 7:**
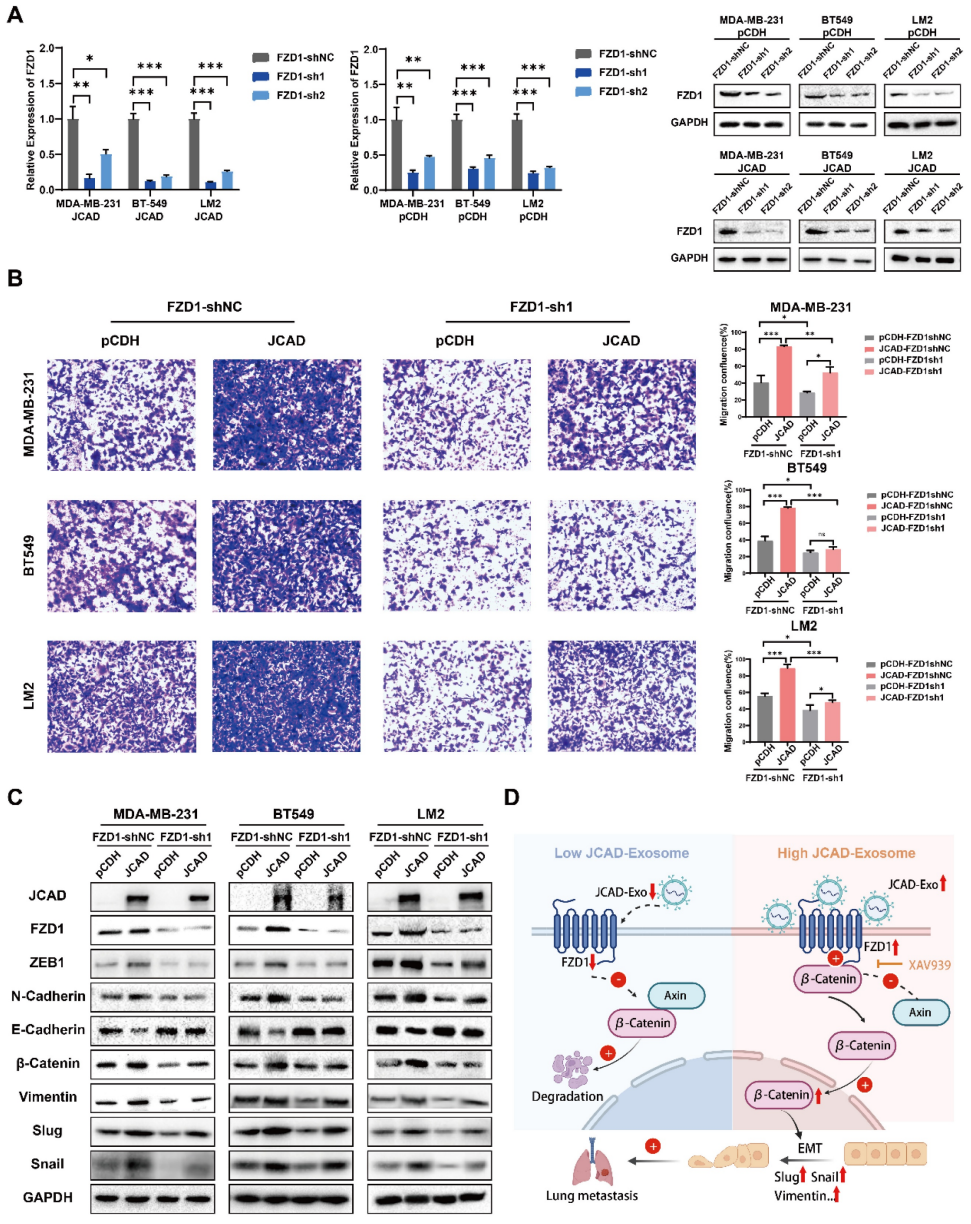
** JCAD activates the Wnt/β-catenin pathway by promoting an increase in downstream FZD1 expression, which mediates the EMT process and thus promotes the progression of breast cancer. A:** qRT‒PCR and Western blot analysis of FZD1 mRNA and protein levels in stable FZD1-KD JCAD-OE MDA-MB-231, BT549 and LM2 cells and control cells. **B:** Transwell migration experiments verified that interference with FZD1 expression significantly inhibited the migration ability of JCAD-OE MDA-MB-231, BT549 and LM2 cells (10×). **C:** Western blot analysis of the protein levels of key molecules involved in EMT and the Wnt/β-catenin pathway in stable FZD1-KD and FZD1-shNC JCAD-OE MDA-MB-231, BT549 and LM2 cells and control cells. **D:** Schematic diagram of exosomal JCAD promoting the progress of breast cancer.

shRNA technology was used to interfere with FZD1 expression in pCDH and JCAD-OE MDA-MB-231, BT549, and LM2 cells **(Fig. 7A)**. Transwell cell migration assays revealed that JCAD overexpression significantly promoted cell migration in the shNC group. Interfering with FZD1 expression significantly inhibited the JCAD-promoted metastasis phenotype** (Fig. 7B)**. Western blot analysis revealed that JCAD overexpression significantly promoted EMT and activated the Wnt/β-catenin pathway in the shNC group. Interfering with FZD1 expression significantly inhibited the promotion of these pathways by JCAD** (Fig. 7C)**.

Overall, these results suggested that JCAD activates the Wnt/β-catenin pathway by promoting an increase in downstream FZD1 expression, which mediates the EMT process and ultimately promotes breast cancer progression **(Fig. 7D)**.

## Discussion

The present study initially focused on plasma exosomes from patients, confirming that plasma exosomes can be used as liquid biopsy markers to predict breast cancer prognosis. This finding provides a reference for initial diagnosis and prognosis prediction for future patients and sets the stage for validation studies with external cohorts. Continued follow-up and validation of patients in the cohort, combined with data from an external cohort, may lead to the establishment of a prognosis prediction model based on the scoring system. In addition, the present study identified JCAD as an independent prognostic factor of breast cancer that is closely related to stage and prognosis, highlighting it as a key molecule for further study.

The present study revealed that the expression of JCAD in exosomes is consistent with its expression in cells. Moreover, our results demonstrated that JCAD is transferred between breast cancer cells via exosomes and is translated into proteins within the recipient wild-type cell line. These findings support the concept that JCAD is secreted by tumor cells in the form of exosomes, transferred to receptor cells, and subsequently performs its biological function.

Further experiments confirmed that JCAD promotes breast cancer cell proliferation and migration. *In vitro* cell experiments, *in vivo* animal experiments, and analyses at both the cell and exosome levels demonstrated that JCAD exosomes enhance breast cancer cell proliferation, inhibit apoptosis, and increase migration. These findings provide comprehensive evidence that JCAD promotes breast cancer progression and is transferred between tumor cells using exosomes, transferring its biological function to recipient cells.

Mechanistically, the present study revealed that JCAD activates the Wnt/β-catenin pathway by increasing downstream FZD1 expression, thereby mediating the EMT process and promoting breast cancer progression. Further studies are needed to elucidate the detailed mechanisms by which JCAD upregulates FZD1. Previous studies have indicated that the Wnt/β-catenin signaling pathway is inhibited by secreted frizzled-related proteins (SFRPs), which act as endogenous inhibitors. Methylation of SFRP genes prevents them from exerting their antitumor effects, leading to abnormal activation of the Wnt signaling pathway and promoting tumor development 33. SFRPs interact with FZDs, and MBD2 alternative splicing inhibits FZD1 activation under hypoxic conditions, thereby promoting breast cancer metastasis 34. Thus, the methylation status and regulation of SFRPs and FZDs are crucial for the function of this pathway. Future studies should investigate whether JCAD affects the regulation of FZD1.

In addition to its role in breast cancer, the overexpression of FZD1 attenuated the increase in cell apoptosis and decrease in cell proliferation in imatinib-resistant renal cell carcinoma cells 35 and the drug that inhibits the Wnt signaling pathway suppresses the expression of FZD1 and promotes the cell death in colorectal cancer 36. Besides, it has been shown that the abnormal expression of FZD receptors contributes to the manifestation of malignant characteristics in human tumors such as enhanced cell proliferation, metastasis and chemotherapy resistance 37. Since JCAD, as an upstream of FZD1, is closely related to breast cancer prognosis and exosomes play a role in tumor resistance, investigating this signaling pathway and its implications for drug resistance in breast cancer is highly important.

The present study highlighted the potential of plasma-derived exosomes as prognostic markers in breast cancer and explored, for the first time, the role of exosomal JCAD in promoting breast cancer progression. In addition, XAV939 can be used as a potential therapeutic approach to provide a therapeutic direction for subsequent JCAD overexpression breast cancer. We aim to continue to follow patient prognoses and build rigorous prognosis prediction models incorporating data from external cohorts, and dedicated to the clinical value research of β-catenin inhibitors such as XAV939. Although the present study did not fully elucidate the mechanisms by which JCAD regulates FZD1 and the exploration of exocrine levels is still in its infancy, the present findings provide a basis for further investigations of JCAD function in breast cancer. Recent research on JCAD in tumors, particularly in breast cancer, is limited. Therefore, further in-depth exploration of JCAD holds great promise but also poses significant challenges.

## Supplementary Material

Supplementary figures.

## Figures and Tables

**Table 1:** Clinical and pathological characteristics of the 111 patients with breast cancer in the present study

Clinical and pathological characteristics	Stage I (%) N=26	Stage II (%) N=36	Stage III (%) N=15	Stage IV (%) N=34	*P* value
**Age (years)**					0.105
< 50	15 (57.7)	18 (50.0)	5 (33.3)	10 (29.4)	
≥ 50	11 (42.3)	18 (50.0)	10 (66.7)	24 (70.6)	
**T**					< 0.001
T1	26 (100.0)	6 (16.7)	0	4 (11.8)	
T2	0	30 (83.3)	5 (33.3)	15 (44.1)	
T3	0	0	7 (46.7)	7 (20.6)	
T4	0	0	3 (20.0)	8 (23.5)	
**N**					< 0.001
Negative	26 (100.0)	10 (27.8)	1 (6.7)	1 (2.9)	
Positive	0	26 (72.2)	14 (93.3)	33 (97.1)	
**M**					< 0.001
Negative	26 (100.0)	36 (100.0)	15 (100.0)	0	
Positive	0	0	0	33 (100.0)	
**ER**					0.074
Negative	8 (30.8)	23 (63.9)	7 (46.7)	15 (44.1)	
Positive	18 (69.2)	13 (36.1)	8 (53.3)	19 (55.9)	
**PR**					0.236
Negative	11 (42.3)	24 (66.7)	10 (66.7)	19 (55.9)	
Positive	15 (57.7)	12 (33.3)	5 (33.3)	15 (44.1)	
**HER2**					0.005
Negative	22 (84.6)	15 (41.7)	10 (66.7)	17 (50.0)	
Positive	4 (15.4)	21 (58.3)	5 (33.3)	17 (50.0)	
**Ki67**					0.016
≤ 20%	14 (53.8)	8 (22.2)	2 (13.3)	9 (26.5)	
>20%	12 (46.2)	28 (77.8)	13 (86.7)	25 (73.5)	
**Immunohistochemical type**					0.129
Luminal	18 (69.2)	13 (36.1)	8 (53.3)	19 (55.9)	
HER2 positive	2 (7.7)	12 (33.3)	3 (20.0)	10 (29.4)	
TNBC	6 (23.1)	11 (30.6)	4 (26.7)	5 (14.7)	

**Table 2:** Prognosis-related differentially expressed genes and coefficients identified through LASSO regression analysis of 11 differentially expressed genes

Gene	Coefficient
IGFBP5	7.69×10^-5^
JCAD (KIAA1462)	6.75×10^-6^
MAP1B	3.71×10^-4^
MGP	1.67×10^-4^
VASH1	1.01×10^-4^

**Table 3:** Clinical and pathological characteristics of 119 patients with breast cancer in the present validation cohort

Clinical and pathological characteristics	Tis/Stage I (%) N=34	Stage II (%) N=22	Stage III (%) N=20		Stage IV (%) N=43	*P* value
**Age (years)**						0.870
< 50	17 (50.0)	9 (40.9)	10 (50.0)		22 (51.2)	
≥ 50	17 (50.0)	13 (59.1)	10 (50.0)		21 (48.8)	
**T**						< 0.001
Tis	5 (14.7)	0	0		0	
T1	29 (85.3)	4 (18.2)	0		10 (23.3)	
T2	0	17 (77.3)	1 (5.0)		25 (58.1)	
T3	0	1 (4.5)	13 (65.0)		3 (7)	
T4	0	0	6 (30.0)		5 (11.6)	
**N**						< 0.001
Negative	34 (100.0)	2 (9.1)	1 (5.0)		12 (27.9)	
Positive	0	20 (90.9)	19 (95.0)		31 (72.1)	
**M**						< 0.001
Negative	34 (100.0)	22 (100.0)	20(100.0)		0	
Positive	0	0	0		43(100.0)	
**ER**						< 0.001
Negative	6 (17.6)	5 (22.7)		8 (40.0)	33 (76.7)	
Positive	28 (82.4)	17 (77.3)		12 (60.0)	10 (23.3)	
**PR**						< 0.001
Negative	6 (17.6)	8 (36.4)		9 (45.0)	35 (81.4)	
Positive	28 (82.4)	14 (63.6)		11 (55.0)	8 (18.6)	
**HER2**						0.002
Negative	31 (91.2)	13 (59.1)		15 (75.0)	40 (93.0)	
Positive	3 (8.8)	9 (40.9)		5 (25.0)	3 (7.0)	
**Ki67**						0.002
≤ 20%	16 (47.1)	3 (13.6)		4 (20.0)	5 (11.6)	
> 20%	18 (52.9)	19 (86.4)		16 (80.0)	38 (88.4)	
**Immunohistochemical type**						< 0.001
Luminal	27 (79.4)	10 (45.5)		10 (50.0)	9 (20.9)	
HER2 positive	3 (8.8)	9 (40.9)		5 (25.0)	3 (7.0)	
TNBC	4 (11.8)	3 (13.6)		5 (25.0)	31 (72.1)	
						

**Table 4:** Baseline clinical and pathological information of the JCAD high- and low-expression groups in the present study cohort

Clinical and pathological characteristics	Total (%) N=111	JCAD expression		*P* value
		Low (%) N=55	High (%) N=56	
**Age (years,** 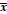 **±s)**		50.1±9.1	52.5±9.4	0.159
**Age (years)**				0.218
< 50	48 (43.2)	27 (49.1)	21 (37.5)	
≥ 50	63 (56.8)	28 (50.9)	35 (62.5)	
**T**				0.380
T1	32 (28.8)	20 (36.4)	12 (21.4)	
T2	53 (47.7)	23 (41.8)	30 (53.6)	
T3	15 (13.5)	7 (12.7)	8 (14.3)	
T4	11 (9.9)	5 (9.1)	6 (10.7)	
**N**				0.552
Negative	38 (34.2)	19 (34.5)	19 (33.9)	
Positive	73 (65.8)	36 (65.5)	37 (66.1)	
**M**				**0.005**
M0	77 (69.4)	45 (81.8)	32 (57.1)	
M1	34 (30.6)	10 (18.2)	24 (42.9)	
**Stage**				**0.037**
I	26 (23.4)	15 (27.3)	11 (19.6)	
II	36 (32.4)	20 (36.4)	16 (28.6)	
III	15 (13.5)	10 (18.2)	5 (8.9)	
IV	34 (30.6)	10 (18.2)	24 (42.9)	
**ER**				0.509
Negative	53 (47.7)	28 (50.9)	25 (44.6)	
Positive	58 (52.3)	27 (49.1)	31 (55.4)	
**PR**				0.785
Negative	64 (57.7)	31 (56.4)	33 (58.9)	
Positive	47 (42.3)	24 (43.6)	23 (41.1)	
**HER2**				0.379
Negative	64 (57.7)	34 (61.8)	30 (53.6)	
Positive	47 (42.3)	21 (38.2)	26 (46.4)	
**Immunohistochemical type**				0.371
Luminal	58 (52.3)	27 (49.1)	31 (55.4)	
HER2 positive	27 (24.3)	12 (21.8)	15 (26.8)	
TNBC	26 (23.4)	16 (29.1)	10 (17.9)	

**Table 5:** Univariate Cox regression analysis of the clinicopathological factors and DFS of breast cancer patients

Covariates	Univariate analysis	
	HR (95% CI)	*P* value
**DFS**		
**MAP1B expression (Low vs. High)**	4.175 (1.664-10.478)	**0.002**
**JCAD expression (Low vs. High)**	4.424 (1.653-11.838)	**0.003**
**VASH1 expression (Low vs. High)**	3.614 (1.500-8.711)	**0.004**
**MGP expression (Low vs. High)**	2.043 (0.895-4.664)	0.090
**IGFBP5 expression (Low vs. High)**	1.917 (0.841-4.373)	0.122
**Age (<50 years vs.≥50 years)**	4.383 (1.503-12.787)	**0.007**
**T**		0.199
T1 vs. T2	1.621 (0.517-5.084)	0.408
T1 vs. T3	2.926 (0.730-11.728)	0.130
T1 vs. T4	3.856 (0.941-15.793)	0.061
**N (N0 vs. N1)**	3.289 (1.121-9.653)	**0.030**
**M (M0 vs. M1)**	98.513 (12.497-776.574)	**<0.001**
**Stage**		**0.001**
**ER (Negative vs. Positive)**	0.740 (0.336-1.632)	0.456
**PR (Negative vs. Positive)**	0.626 (0.270-1.455)	0.276
**Her-2 (Negative vs. Positive)**	1.436 (0.655-3.150)	0.366
**Immunohistochemical type**		0.569
Luminal vs. TNBC	0.905 (0.334-2.455)	0.845
Her-2 positive vs. TNBC	1.467 (0.508-4.239)	0.478

**Table 6:** Multivariate Cox regression analysis of the clinicopathological factors and DFS of breast cancer patients

Covariates	Multivariate analysis	
	HR (95% CI)	*P* value
**DFS**		
**JCAD expression (Low vs. High)**	4.455 (1.446-13.727)	**0.009**
**M (M0 vs. M1)**	134.240 (14.819-1216.064)	**< 0.001**
