# JCAD-Derived from Plasma Exosomes: Promoting Tumor Cell Progression and Predicting Poor Outcomes in Breast Cancer: Erratum

**DOI:** 10.7150/ijbs.134047

**Published:** 2026-10-12

**Authors:** Douwaner Liu, Min Xiong, Xiaoting Chen, Xuliren Wang, Yuting Sang, Shiyang Liu, Liyi Zhang, Weiru Chi, Hengyu Ren, Bingqiu Xiu, Qi Zhang, Yayun Chi, Jiong Wu, Jingyan Xue

**Affiliations:** 1 Key Laboratory of Breast Cancer in Shanghai, Department of Breast Surgery, Fudan University Shanghai Cancer Center, Shanghai 200032, China.; 2 Department of Radiation Oncology, Huadong Hospital, Fudan University, Shanghai 200040, China.; 3 Department of Oncology, Shanghai Medical College, Fudan University, Shanghai 200032, China.

In the process of checking the raw data, we noticed several inadvertent mistakes occurring in Figure 3, Figure 6 and Supplementary Figure 6. The detailed information is as follows.

In Figure 3D, during the preparation of the panels, the representative images of JCAD and SH1 group of LM2 cell line were pasted and placed by mistake. In Figure 3E, the representative images of SH2 group of BT549 cell line was mistakenly pasted and placed. In addition, the JCAD bands in Figure 6C and FZD1 bands in Figure 6J had been validated in Figure 2A, 2B and 6I. To avoid any potential misunderstandings or redundancies, we decided to remove these bands from the Figure 6C and 6J. Moreover, the representative bands of N-Cadherin expression in LM2-OE group and GAPDH expression in LM2-KD group in Figure 6C, as well as the representative bands of GAPDH expression in BT549 and LM2 cell lines in Figure 6I were mistakenly pasted and placed. Finally, in Supplementary Figure 6D, the representative image of FZD1+DMSO group of MDA-MB-231 cell line was mistakenly pasted and placed.

The correct results should be as shown below. We sincerely apologize for these oversights and declare that these corrections will not affect the description, interpretation, or conclusions detailed in the original paper.

## Figures and Tables

**Figure 3:**
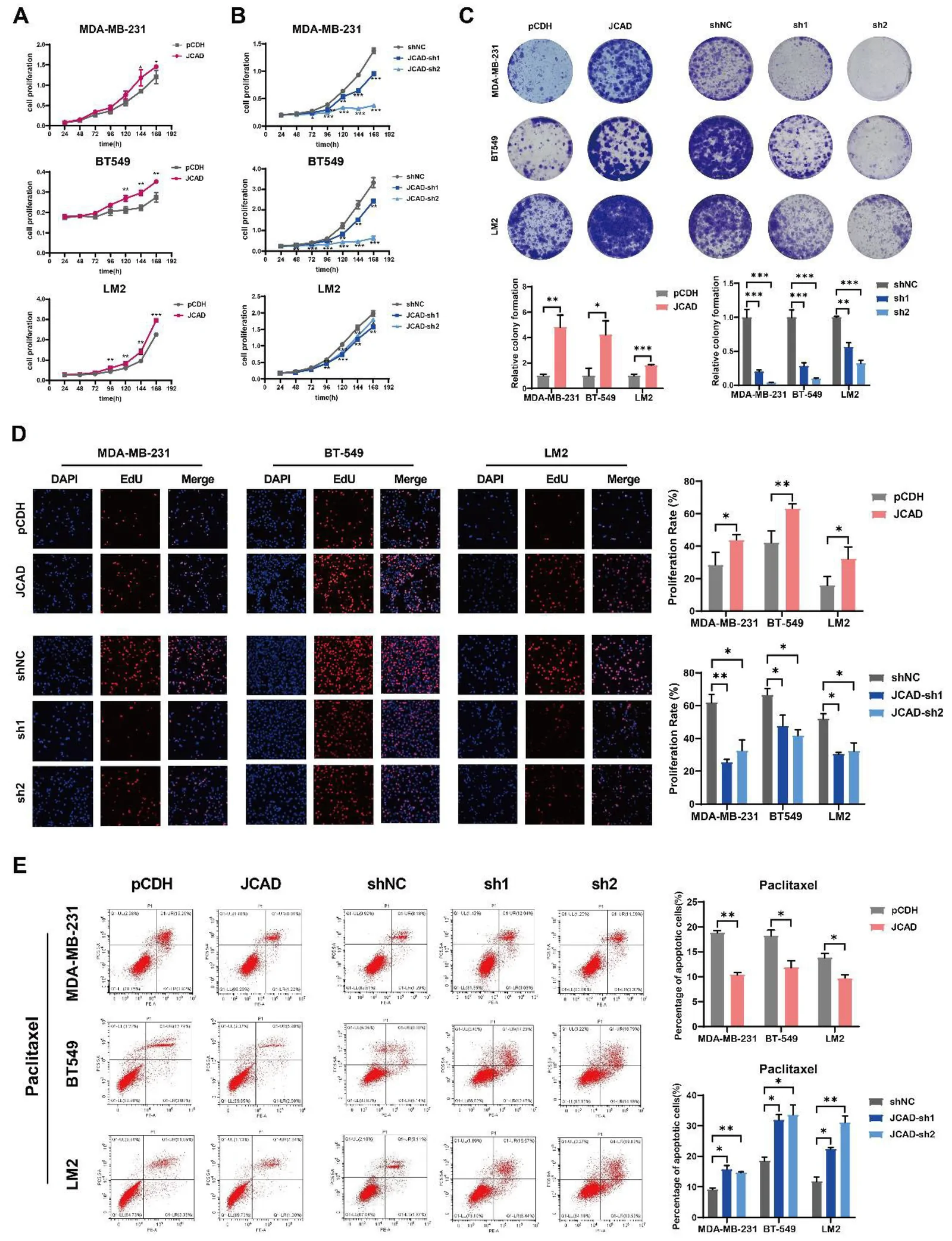
JCAD promotes the proliferation of breast cancer cells.

**Figure 6:**
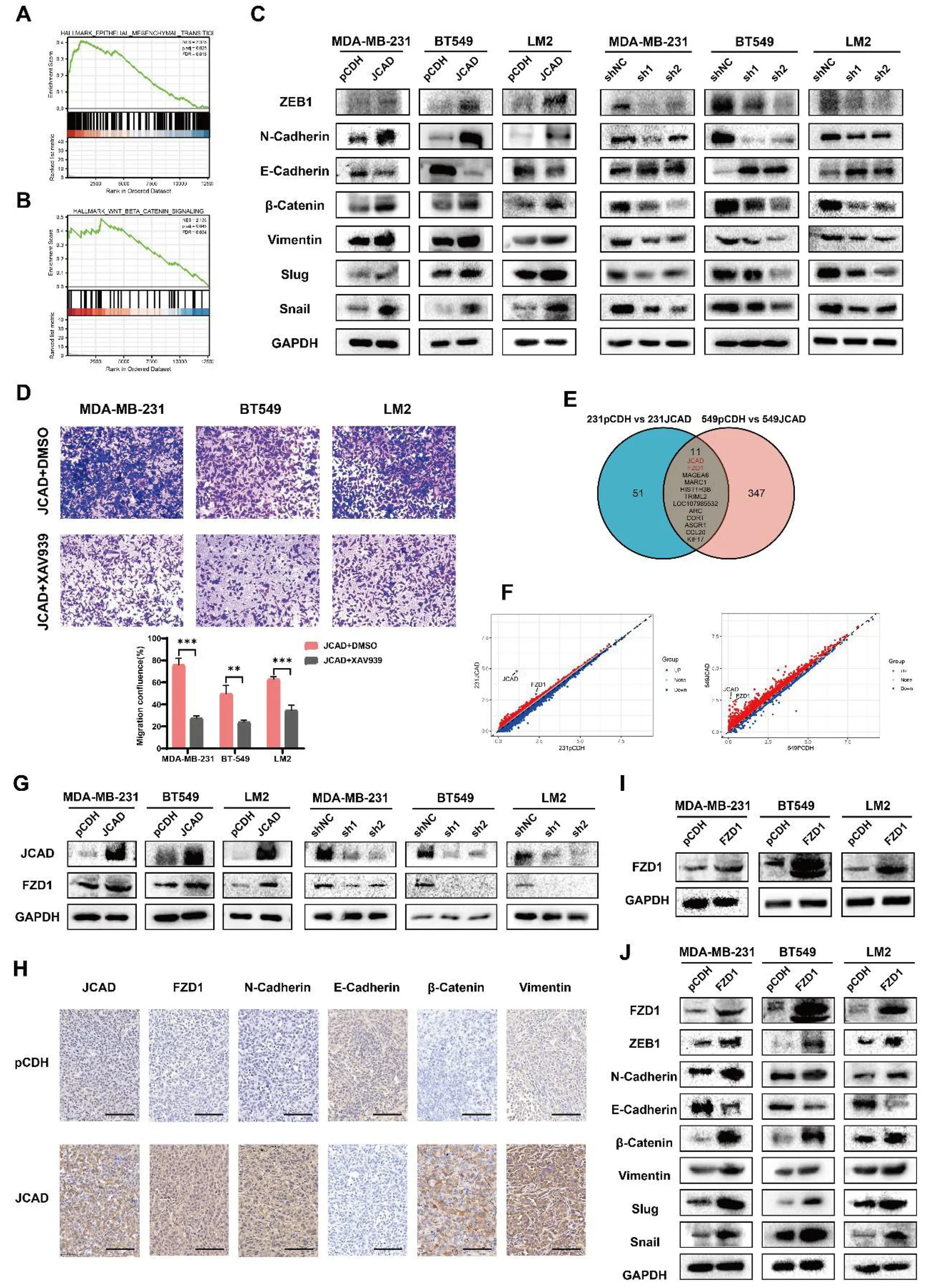
JCAD activates EMT, activates the Wnt/β-catenin pathway and induces high expression of FZD1.

**Figure A:**
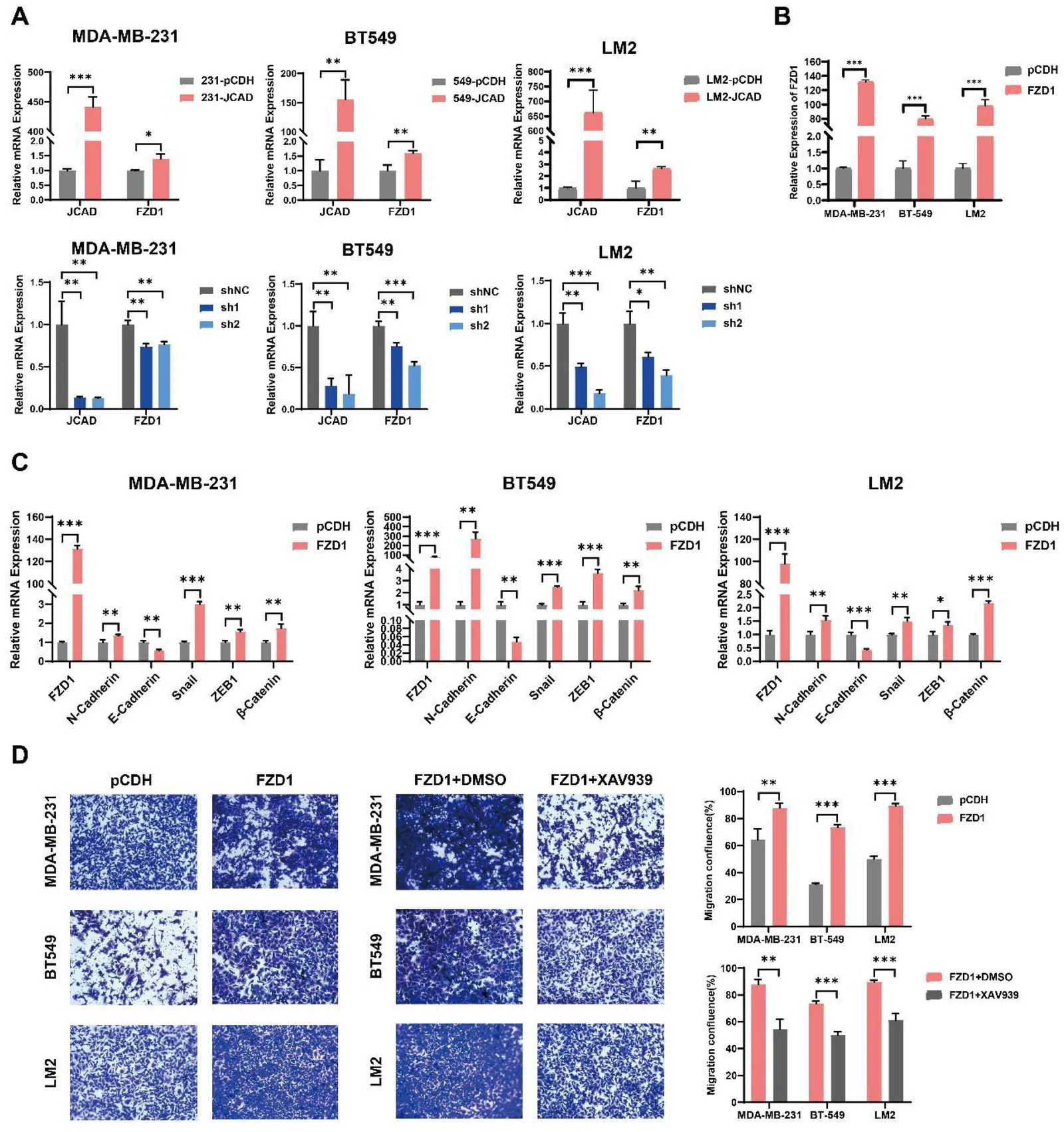
Supplementary Figure 6: JCAD induces high expression of FZD1.

