# Supplementary material for: JCAD-Derived from Plasma Exosomes: Promoting Tumor Cell Progression and Predicting Poor Outcomes in Breast Cancer
Source: Int J Biol Sci. 2025 Jul 25;21(11):4816–33. doi: 10.7150/ijbs.107763 (PMC12374822; doi:10.7150/ijbs.107763)
Supplement: Supplementary file 1 — Supplementary figures. [file ijbsv21p4816s1.pdf]

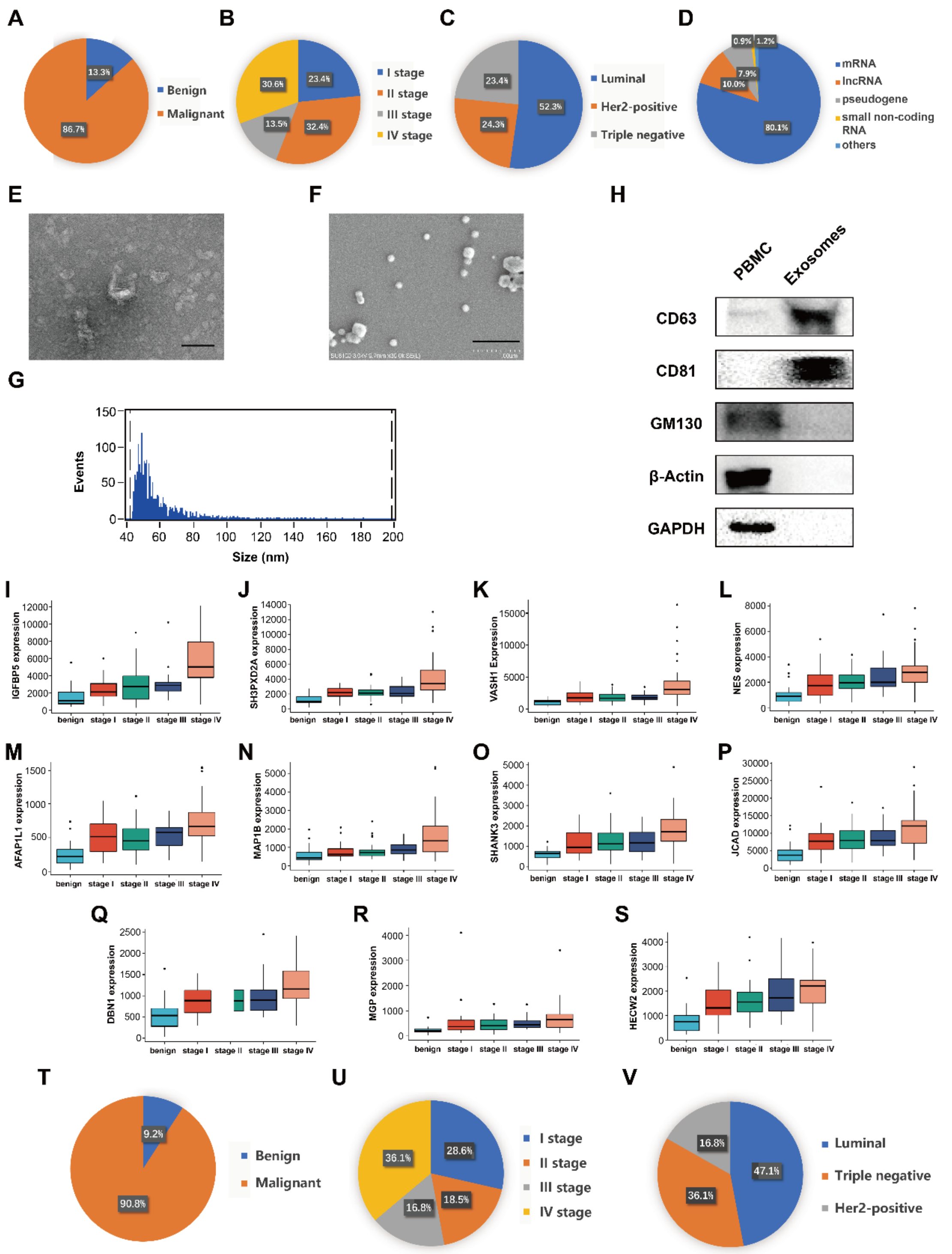

Supplementary Figure 1: Collection and validation of plasma exosomes and screening of differentially expressed genes. A: The proportion of benign and malignant patients among 128 patients; **B:** The proportion of malignant patients in different stages. **C:** The proportion of different subtypes of malignant patients. **D:** The proportion of different gene types in sequencing results. **E:** Plasma exosomes extracted

by the kit under transmission electron microscopy (scale=100nm in the figure). **F:** Plasma exosomes extracted by the kit under scanning electron microscopy (scale=1μm in the figure). **G:** Nano particle analyzer (NTA) was used to analyze the particle size range of plasma exosomes. **H:** The protein expression levels of CD63, CD81, GM130, β-Actin and GAPDH. **I-S:** Box plots of 11 differentially expressed genes with an increasing trend with stage. **T:** The proportion of benign and malignant patients in the validation cohort; **U:** The proportion of malignant patients in each stage of the validation cohort; **V:** The proportion of malignant patient subtypes in the validation cohort.

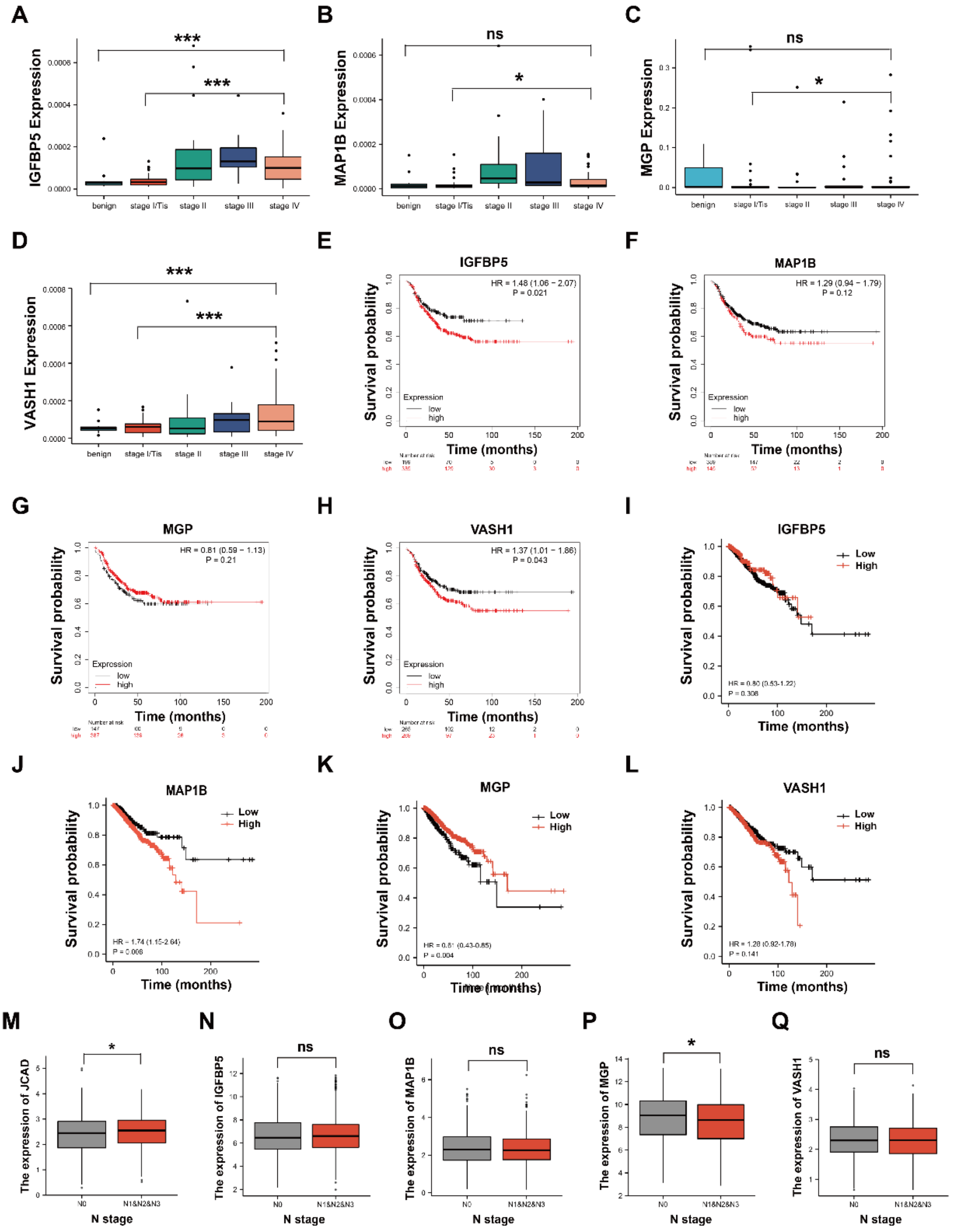

Supplementary Figure 2: The relationship between IGFBP5, MAP1B, MGP, VASH1 and staging and prognosis. A-D: Box plots of IGFBP5, MAP1B, MGP and VASH1 expression levels in patients of different stages in the validation cohort. **E-H:** The Kaplan-Meier survival curves of IGFBP5, MAP1B, MGP and VASH1 in the Kaplan Meier Plotter public database. **I-L:** The Kaplan-Meier survival curves of IGFBP5, MAP1B, MGP and VASH1 in the TCGA public database. **M-Q:** Box plots of

the expression levels of JCAD, IGFBP5, MAP1B, MGP and VASH1 in the TCGA database for patients with or without lymph node metastasis.

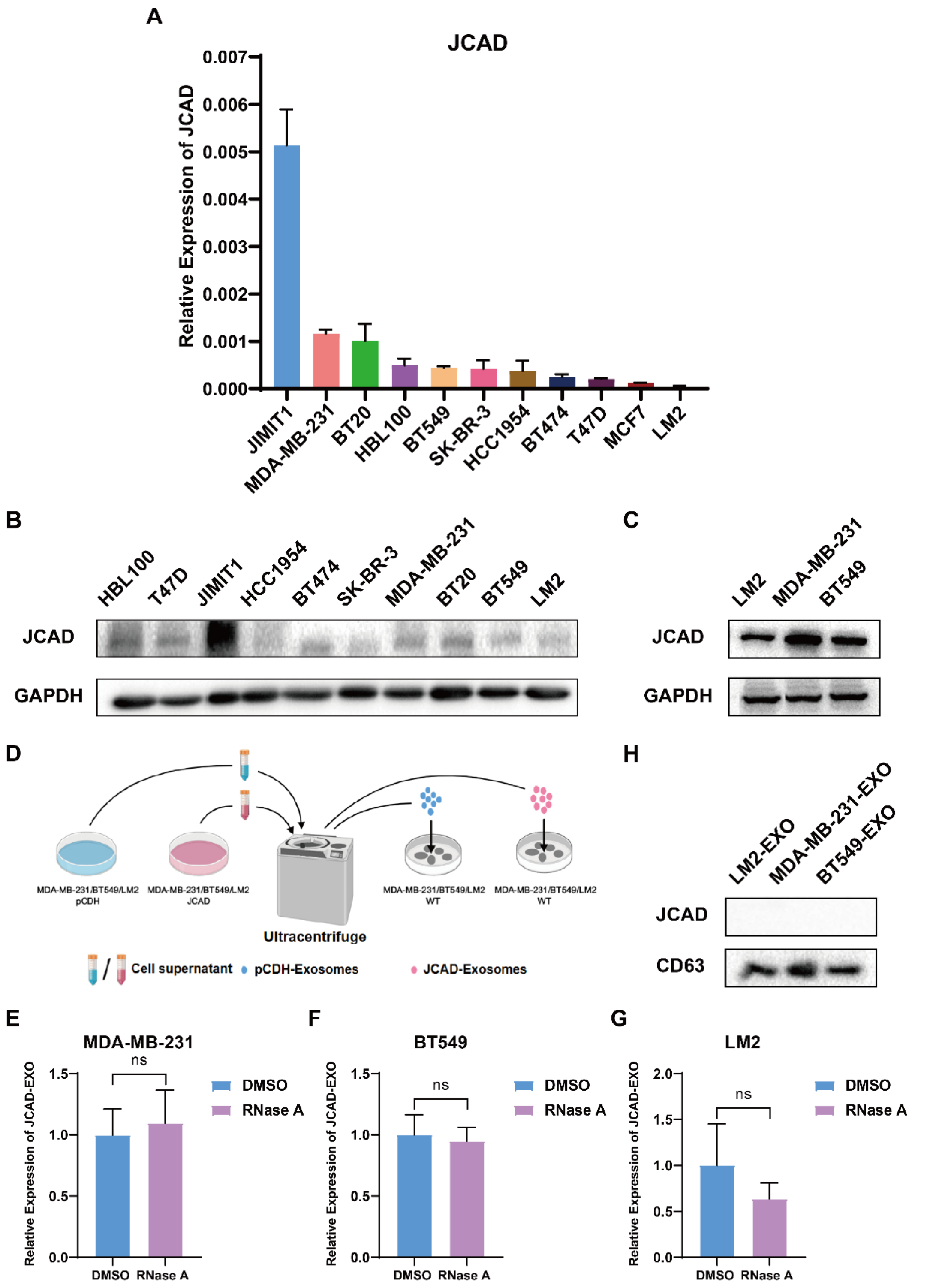

Supplementary Figure 3: The expression of JCAD in breast cancer cells and the schematic diagram of exosome experiments. A: qRT-PCR analysis of JCAD mRNA levels in breast cancer cell lines. **B:** Western blot analysis of JCAD protein levels in breast cancer cell lines. **C:** Western blot analysis of JCAD protein levels in LM2, MDA-

MB-231 and BT549. **D:** Schematic diagram of extracting exosomes from cell supernatant by ultracentrifugation and adding them to wild-type cell lines for culture. **E-G:** The expression level of JCAD in exosomes derived from JCAD-OE MDA-MB-231, BT549, and LM2 cells cannot be inhibited by RNase A. **H:** Western blot analysis of JCAD and CD63 protein levels in exosomes of LM2, MDA-MB-231 and BT549 cells.

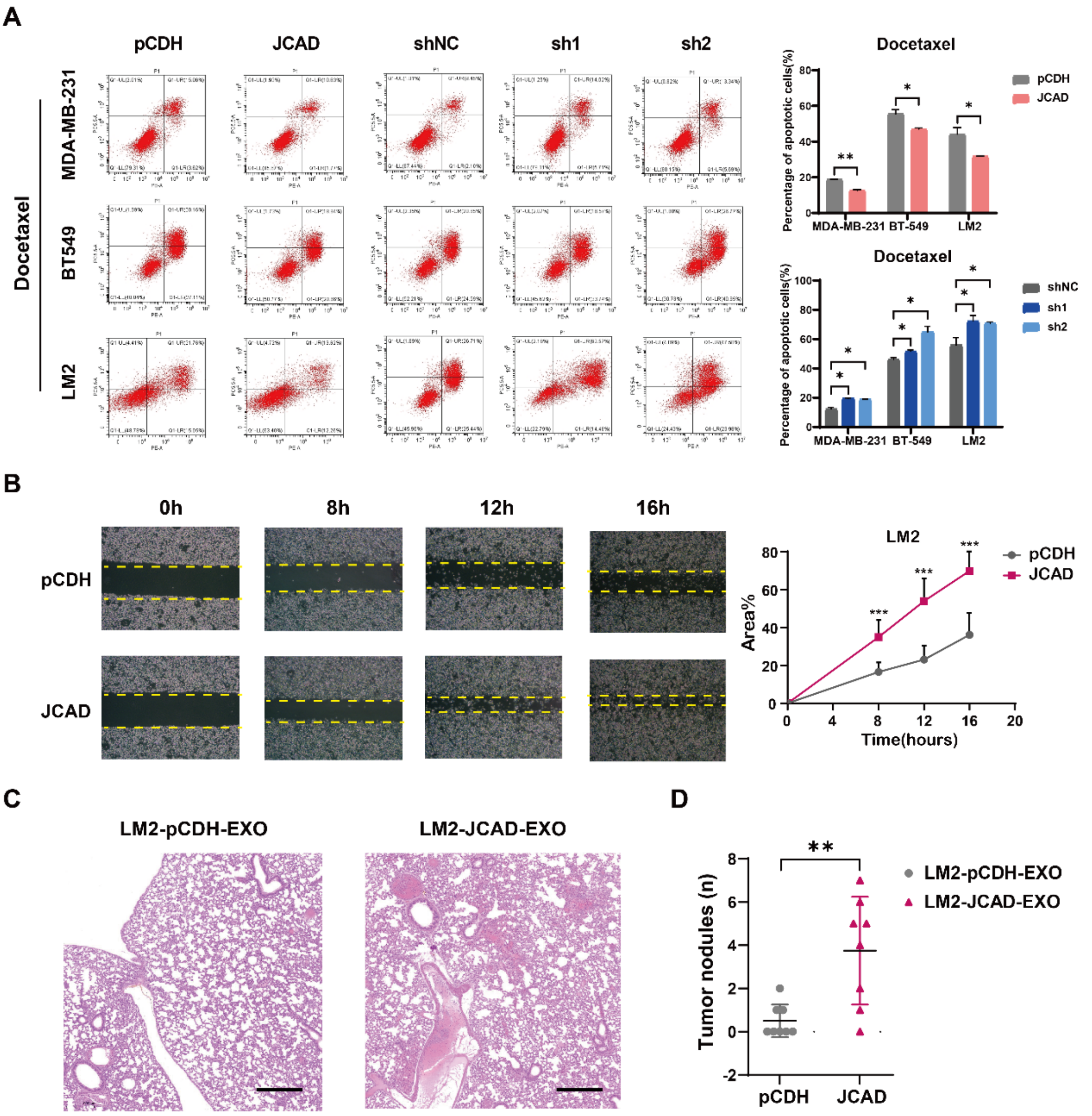

Supplementary Figure 4: JCAD inhibits the apoptosis and promotes the metastasis of breast cancer cells and exosomal JCAD promotes lung metastasis in vivo. A: Induction of apoptosis by adding docetaxel verified the apoptosis of JCAD-OE and JCAD-KD MDA-MB-231, BT549, and LM2 cells compared with control cells. **B:** Scratch assay verified the migration of JCAD-OE LM2 cells compared with control cells. **C:** HE staining of lung metastatic nodules of the pCDH-EXO group and JCAD-EXO group (HE: hematoxylin and eosin; scale bar=500 μm). **D:** Statistics of lung metastatic nodule counts of the pCDH-EXO group and JCAD-EXO group.

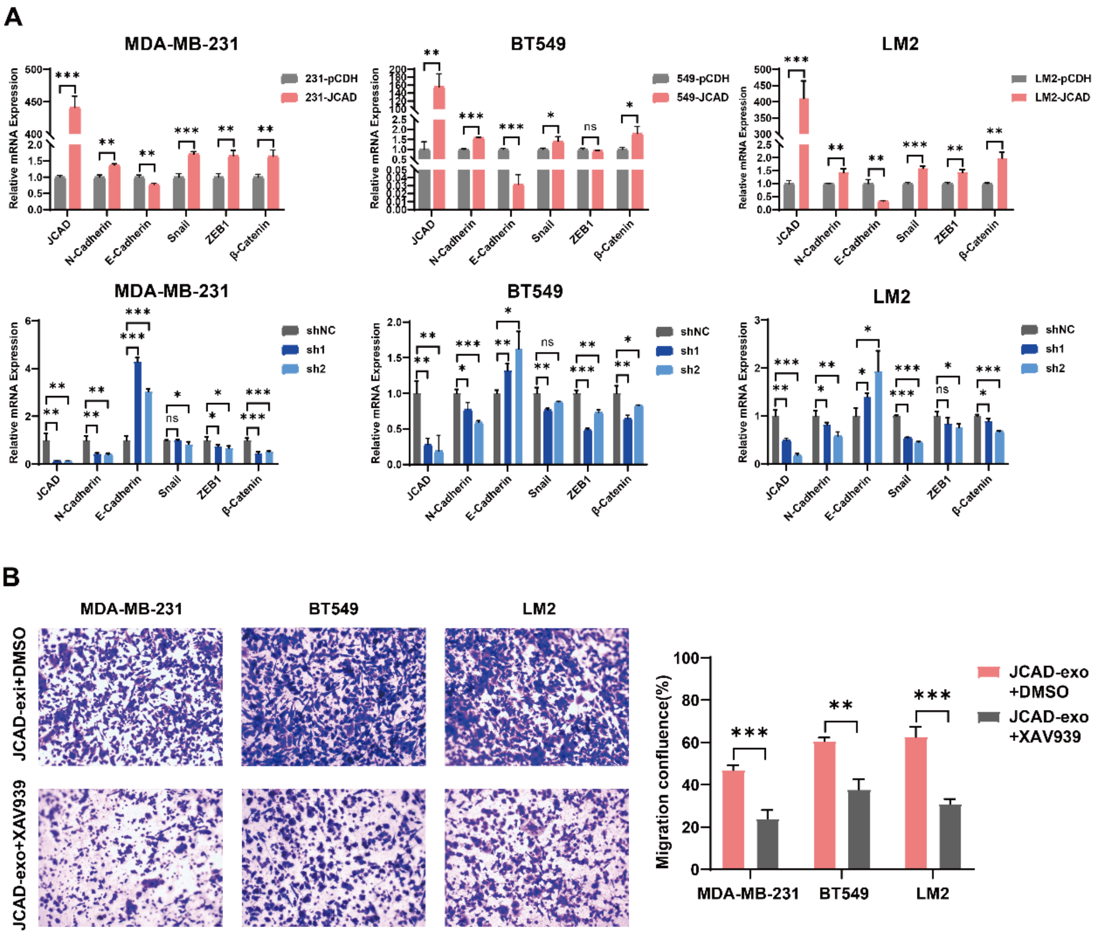

Supplementary Figure 5: JCAD activated EMT and Wnt/β-Catenin pathway. A: qRT-PCR analysis of key molecules mRNA levels of EMT and Wnt/β-Catenin pathway in JCAD-OE and JCAD-KD MDA-MB-231, BT549 and LM2 cells and control cells. **B:** After culturing the exosomes of JCAD OE MDA-MB-231, BT549 and LM2 cells with corresponding wild-type cell lines, transwell cell migration experiment verified that β-Catenin inhibitor XAV939 can reduce the migration ability of exosomal JCAD (10×).

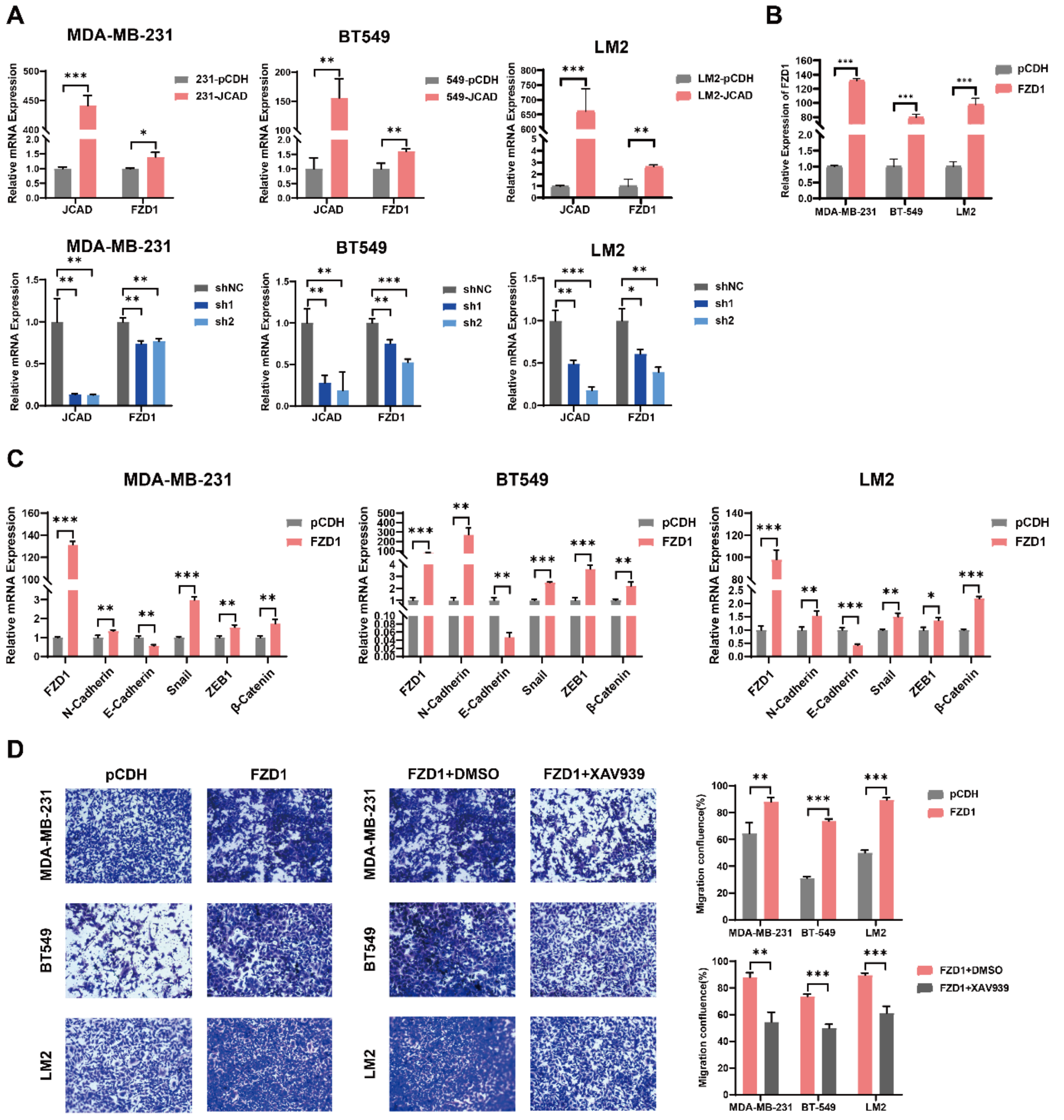

Supplementary Figure 6: JCAD induces high expression of FZD1. A: qRT-PCR analysis of FZD1 mRNA levels in JCAD-OE and JCAD-KD MDA-MB-231, BT549 and LM2 cells and control cells. **B:** qRT-PCR analysis of FZD1 mRNA levels in stable FZD1-OE MDA-MB-231, BT549 and LM2 cells and control cells. **C:** qRT-PCR analysis of key molecules mRNA levels of EMT and Wnt/β-Catenin pathway in FZD1-OE MDA-MB-231, BT549 and LM2 cells and control cells. **D:** Transwell cell migration experiment verified that FZD1-OE MDA-MB-231, BT549 and LM2 cells can promote migration of breast cancer cells and β-Catenin inhibitor XAV939 can reduce this ability (10×).
